# Astrocytes‐derived extracellular vesicles in motion at the neuron surface: Involvement of the prion protein

**DOI:** 10.1002/jev2.12114

**Published:** 2021-07-12

**Authors:** Giulia D'Arrigo, Martina Gabrielli, Federica Scaroni, Paolo Swuec, Ladan Amin, Anna Pegoraro, Elena Adinolfi, Francesco Di Virgilio, Dan Cojoc, Giuseppe Legname, Claudia Verderio

**Affiliations:** ^1^ Department of Neuroscience Scuola Internazionale Superiore di Studi Avanzati (SISSA) Trieste Italy; ^2^ Institute of Neuroscience CNR National Research Council of Italy Milano Italy; ^3^ Department of Biosciences Università degli Studi di Milano Milano Italy; ^4^ Centro di Ricerca Pediatrica Romeo ed Enrica Invernizzi Università degli Studi di Milano Milano Italy; ^5^ Department of Medical Sciences Section of Experimental medicine Università degli Studi di Ferrara Ferrara Italy; ^6^ Institute of Materials CNR National Research Council of Italy Area Science Park – Basovizza Trieste Italy

**Keywords:** astrocytes, cytoskeleton, extracellular vesicles, neurons, optical tweezers, prion protein, PrP knock‐out

## Abstract

Astrocytes‐derived extracellular vesicles (EVs) are key players in glia‐neuron communication. However, whether EVs interact with neurons at preferential sites and how EVs reach these sites on neurons remains elusive. Using optical manipulation to study single EV‐neuron dynamics, we here show that large EVs scan the neuron surface and use neuronal processes as highways to move extracellularly. Large EV motion on neurites is driven by the binding of EV to a surface receptor that slides on neuronal membrane, thanks to actin cytoskeleton rearrangements. The use of prion protein (PrP)‐coated synthetic beads and PrP knock out EVs/neurons points at vesicular PrP and its receptor(s) on neurons in the control of EV motion. Surprisingly, a fraction of large EVs contains actin filaments and has an independent capacity to move in an actin‐mediated way, through intermittent contacts with the plasma membrane. Our results unveil, for the first time, a dual mechanism exploited by astrocytic large EVs to passively/actively reach target sites on neurons moving on the neuron surface.

**Abbreviations**
Aβ: amyloid‐beta
CAR: coxsackievirus adenovirus receptor
CytoD: cytochalasin D
EVs: extracellular vesicles
KRH: Krebs‐Ringer's HEPES solution
lEVs: large extracellular vesicles
NDs: neurodegenerative diseases
OT: optical tweezers
PAR4: prostate apoptosis response 4
PrP: prion protein
sEVs: small extracellular vesicles
SOD1: superoxide dismutase 1
TNTs: tunneling nanotubes
TRPS: tunable resistive pulse sensing

## 1 INTRODUCTION

Extracellular vesicles (EVs) are circular membrane fragments released by all cells which function as intercellular signalling vehicles. EVs influence the behaviour of target cells in multiple ways, including the transfer of bioactive cargoes and the activation of signalling events at the cell surface (Holm et al., 2018). In addition, EVs possess enzymatic activity and are capable of modifying the environment surrounding the donor cell by changing the molecular composition of the extracellular fluid (Iraci et al., 2017). EVs were often classified into exosomes, which originate from endocytic pathway, microvesicles or ectosomes, that shed directly from the plasma membrane (Cocucci & Meldolesi, 2015) of healthy cells and apoptotic bodies, which are released by cells undergoing apoptosis. Currently EVs are preferentially distinguished based on physical characteristics (size and density), biochemical composition and cell of origin (Thery et al., 2018), due to difficulties in isolating EVs on the basis of their mechanisms of biogenesis. Small EVs (sEVs) are < 100–200 nm in size, while medium/large EVs range from 200 to 1,000 nm in diameter (Thery et al., 2018). Despite small EVs (exosomes) were the most widely studied EV population, conveying the false impression to be more important than other EV types (Tkach et al., 2018), the need is emerging to comprehensively study all type of secreted EVs to fully define the activity of these highly heterogeneous structures.

Compelling evidence indicates that astrocytes can communicate with neurons in the brain through secretion of EVs (Budnik et al., 2016; Holm et al., 2018; Kramer‐Albers & Hill, 2016). Astrocyte‐derived EVs stimulate excitatory transmission (Antonucci et al., 2012) and contain neuroprotective and growth promoting proteins, such as Hsp/Hsc70 (Taylor et al., 2007) and synapsin I (Wang et al., 2011). However, in the diseased brain astrocytes‐derived EVs become vehicle of pathogenic molecules, for example, a set of microRNAs that impair synaptic stability (Prada et al., 2018) or suppress neuronal differentiation and neurite outgrowth (Chaudhuri et al., 2018), the inflammatory cytokine IL‐1β (Bianco et al., 2009), the pro‐apoptotic agents ceramide and PAR4 (prostate apoptosis response 4) (Wang et al., 2012), complement factors (Goetzl et al., 2018) and misfolded proteins associated with neurodegenerative diseases (NDs) such as amyloid‐beta (Aβ), tau and superoxide dismutase 1 (SOD1) (Chiarini et al., 2017; Goetzl et al., 2016; Sollvander et al., 2016). Importantly, packaging into EVs appears to partly mediate transynaptic spread of misfolded proteins, which propagate throughout several brain areas over time in patients with NDs (Basso & Bonetto, 2016). While recent evidence shows that tau/Aβ‐containing small EVs released by primary neurons or isolated from Alzheimer patients' brains can be internalized and transferred between neurons through axonal projections (Polanco et al., 2018; Sardar Sinha et al., 2018; Wang et al., 2017), how large EVs move across the synapse and reach distant target cells remains elusive.

In this study we focused on this less studied population of large EVs, whose interaction with neurons can be monitored through bright field microscopy. Exploiting optical manipulation to study single EV‐neuron dynamics, we show that most large EVs move on the neuron surface in anterograde or retrograde directions, and provide a dual mechanism for EV motion. In particular, we show that the majority of large EVs exploits the prion protein (PrP) and its neuronal receptors to passively navigate along neuronal projections while a minor fraction of EVs has an independent capacity to move on the neuron surface. These findings represent first evidence for the existence of a regulated trafficking of large EVs outside the neuron surface and provide initial evidence on the molecular mechanism controlling large EV motion.

## 2 MATERIALS AND METHODS

### 2.1 Primary cultures and treatment

All the experimental procedures to establish primary cultures followed the guidelines defined by the European legislation (Directive 2010/63/EU), and the Italian Legislation (LD no. 26/2014). Astrocytic cultures were established from rat Sprague–Dawley pups (P2) (Charles River, Lecco, Italy), wild type FVB or FVB/ Prnp^0/0^ pups (of either sex) (Lledo et al., 1996). Briefly, after dissection, hippocampi and cortices were dissociated by treatment with trypsin (0.25%, Gibco, Thermo Fisher, Leicestershire, UK) and DNase‐I (Sigma‐Aldrich, St. Louis, MO, USA) for 15 min at 37°C, followed by fragmentation with a pipette. Dissociated cells were plated on poly‐L‐lysine‐coated (Sigma Aldrich, St. Louis, MO, USA) T75 flasks in minimal essential medium (MEM, Invitrogen, Life Technologies, Carlsbad, CA, USA) supplemented with 20% fetal bovine serum (FBS) (Gibco, Life Technologies, Carlsbad, CA, USA) and glucose (5.5 g/L, Sigma Aldrich, St. Louis, MO, USA). To obtain a pure astrocyte monolayer, microglial cells were harvested from 10–14‐day‐old cultures by orbital shaking for 30 min at 200 rpm. Hippocampal neurons were established from the hippocampi of 18‐day‐old foetal Sprague Dawley rats (E18) of either sex (Charles River, Lecco, Italy), wild‐type (wt) FVB and FVB/Prnp^0/0^ mouse embryos of either sex. Briefly, dissociated cells were plated onto poly‐L‐lysine treated coverslips and maintained in Neurobasal with 2% B27 supplement (Invitrogen, Carlsbad, CA, USA), antibiotics, glutamine and glutamate (Sigma Aldrich, St. Louis, MO, USA). Neurons were used at 2–17 DIV (Day‐In‐Vitro). To distinguish axon from dendrites neurons were transfected at DIV15 with a GFP, farnesyl GFP or RFP expressing vector using Lipofectamine 2000 (0.75 μg of plasmid). At this developmental stage the efficiency of transfection is quite low (∼5%) and allows distinction of axons from dendrites (Figure 2m). In a set of experiments, neurons were transfected at 2–3 and 12–15 DIV and imaged 24–48 h later to monitor induction of thin protrusion from transfected neurites. To block cytoskeleton dynamics, neurons were treated with 2 μM Rotenone (Sigma Aldrich, St. Louis, MO, USA) or 1 μM Cytochalasin D (Sigma Aldrich, St. Louis, MO) for 1 h, 100 μM Blebbistatin (Sigma Aldrich, St. Louis, MO, USA) or 10–30 μM Nocodazolo (Sigma Aldrich, St. Louis, MO, USA) for 30 min. Sodium Azide (NaN_3_, Sigma Aldrich, St. Louis, MO, USA) was added at a final 20 mM concentration to acute treatments. To verify inhibition of active lysosome transport in Rotenone‐treated cultures, neurons were loaded with 100 nM LysoTracker DND‐99 (Life Technologies, Carlsbad, CA, USA) for 30 min, washed and imaged by time lapse confocal microscopy. To downregulate PrP in astrocytes or neurons, cells were transfected with PrP‐specific Accell smart pool siRNAs (1:500, Dharmacon, Carlo Erba Reagents Srl, Cornaredo, Italy) using Lipofectamine 2000 (Life Technologies, Carlsbad, CA, USA) for 48 h. Cells were then lysated and PrP‐protein levels evaluated by Western blotting analysis.

### 2.2 EVs isolation treatment and labelling

A total of 15 × 10^6^ astrocytes were exposed to 1 mM ATP (Sigma‐Aldrich, St. Louis, MO, USA) for 30 min in 10 ml of Krebs‐Ringer's HEPES solution (KRH) (125 mM NaCl, 5 mM KCl, 1.2 mM MgSO_4_, 1.2 mM KH_2_PO, 2 mM CaCl_2_, 6 mM D‐glucose, 25 mM HEPES/NaOH, pH 7.4). All conditioned KRH was collected and pre‐cleared from cells and debris by centrifugation at 300 × *g* for 10 min (twice) with a refrigerated centrifuge (ALC 4227 R, rotor ALC 5690). Medium/large EVs were then pelleted from the supernatant by centrifugation at 10,000 × *g* for 30 min with a refrigerated centrifuge (VWR CT15FE, rotor Hitachi T15A61) and resuspended in 150 μl of neuronal medium. EVs were used immediately after isolation by adding 25 μl of the EV suspension to each coverslip of neurons, in a final volume of about 400 μl before optical tweezers manipulation. In a set of experiments, isolated EVs were incubated with 3 μM Cytochalasin D for 1 h at room temperature in 150 μl of neuronal medium before being delivered by optical tweezers. To stain ATP store, EVs were incubated with 1 μM Quinacrine (Sigma Aldrich, St. Louis, MO, USA) for 30 min, washed with KRH and re‐pelleted. The pellet was suspended in 150 μl of KRH and spotted on a glass coverslip for confocal microscopy.

### 2.3 Cytotoxicity assay

Aliquots of conditioned medium (KRH) were collected from 96‐well plated astrocytes maintained in basal conditions or stimulated with ATP for 30 min for the evaluation of lactate dehydrogenase (LDH) activity using the cytotoxicity assay kit (CyQUANT LDH Cytotoxicity Assay Kit, Invitrogen, Carlsbad, CA, USA). LDH levels were measured via a coupled enzymatic assay, which resulted in the formation of a coloured product (Formazan), the absorbance of which was recorded at 490 nm and 680 nm (SPECTROstar Nano, BMG Labtech, Ortenberg, Germany). To measure maximum LDH activity, cells were treated with lysis buffer, according to the manufacturer instructions.

### 2.4 EV characterization by TRPS

Size distribution of medium/large EVs (10,000 × *g* pellet) has been evaluated through Tunable Resistive Pulse Sensing (TRPS) technique, using a qNano instrument (IZON, Christchurch, New Zealand) which provides accurate size measurements of EVs by detecting current blockage from EV passing through a nanopore. Cell supernatant of 7 × 10^6^ astrocytes was partitioned in two aliquots, after clearing from cell debris (300 × *g* × 10 min twice). One aliquot was centrifuged at 10,000 × *g* for 30 min, the pellet resuspended with Izon EV reagent kit and analysed using an NP400 pore. The other aliquot was further centrifuged at 2,000 × *g* for 20 min, to eliminate bigger EVs, before being pelleted at 10,000 × *g*, resuspended and analysed using an NP200. Removal of bigger EVs limited NP200 pore obstruction. Analysis by NP400 pore was used to determine the concentration of EVs > 200 nm, while by NP200 pore we evaluated < 200 nm EV concentration (results were filtered for > 200 nm and < 200 nm respectively).

### 2.5 F‐actin and G‐actin isolation from neurons and EVs

F‐actin/G‐actin ratio was assessed as previously described (Pyronneau et al., 2017). Briefly, cultured neurons or freshly isolated EVs, produced by 75 × 10^6^ astrocytes, were resuspended in cold lysis buffer (10 mM K_2_HPO_4_, 100 mM NaF, 50 mM KCl, 2 mM MgCl_2_, 1 mM EGTA, 0.2 mM DTT, 0.5% Triton X‐100, 1 mM sucrose, pH 7.0) and centrifuged at 15,000 × *g* for 30 min. The G‐actin supernatant was transferred to a fresh tube, and the F‐actin pellet was resuspended in lysis buffer plus an equal volume of a second buffer (1.5 mM guanidine hydrochloride, 1 mM sodium acetate, 1 mM CaCl_2_, 1 mM ATP, 20 mM tris‐HCl, pH 7.5) and then incubated on ice for 1 h to convert F‐actin into soluble G‐actin. Samples were centrifuged at 15,000 × *g* for 30 min, and the supernatant (containing the F‐actin, which was converted to G‐actin) was transferred to a fresh tube. F‐actin and G‐actin samples were loaded with equal volumes and analysed by Western blotting.

### 2.6 Western blotting

Astrocytes and neurons were lysed with a buffer containing 1% SDS, 2 mM EDTA pH 7.4, 10 mM Tris‐HCl pH 7.4 and proteases inhibitor (1:500). A modified version of the Laemmli buffer was then added to a final 1× concentration (15% SDS, 575 mM sucrose, 325 mM Tris‐HCl pH 6.8, 0.5% β‐mercaptoethanol, 0.01% bromo‐phenol blue). EVs released from 20 × 10^6^ astrocytes were lysed with the same Laemmli buffer. 10 μg of total lysates of neurons or astrocytes and the whole pellet of EVs were loaded on a polyacrylamide gel. Proteins were then separated by electrophoresis, blotted on nitrocellulose membrane and probed using mouse anti‐PrP (1:1000; W226; Dr Lothar Stitz, Institute of Immunology, Tuebingen, Germany), rabbit anti‐Alix (1:500; Covalab, Villeurbanne, France), mouse anti‐Flotillin (1:1000, BD Biosciences, CA, USA), rabbit anti‐Annexin A2 (1:5000, Abcam, UK), rabbit anti‐Tom‐20 (1:500; Santa Cruz Biotechnology, CA, USA), mouse anti‐GS28 (1:1000; BD Biosciences, Franklin Lakes, NJ, USA), mouse anti‐Actin (1:500; Sigma, St. Louis, MO, USA), mouse anti‐βIII‐tubulin (1:4000; Promega, Madison, WI, USA) or Rabbit anti‐GAPDH (1:2000; Synaptic Systems, Gottingen, Germany) antibodies. Photographic development was by chemiluminescence (ECL, Euroclone or FEMTO, Thermo Scientific, MA, USA) according to the manufacturer's instructions. Western blot bands were quantified by ImageJ software.

### 2.7 Bead functionalization

1 μm silica beads coated with COOH groups (Kisker‐biotech, Steinfurt, Germany, cat PSi‐1.0 COOH) were functionalized using PolyLink Protein Coupling Kit (Bangs Laboratories Inc., Fishers, IN, USA, cat PL01N) following the manufacturer's protocol. Briefly, about 1.4 × 10^5^ beads were incubated with 1 μg of recombinant full length PrP or C‐terminal of PrP in the presence of 20 mg/ml EDAC for 1 h at room temperature (RT). Functionalized beads were then washed and stored in storage buffer at 4°C. Protein coupling was tested by Immunohistochemistry assay in which coated microspheres were incubated for 1 h with primary anti‐PrP antibodies (W226) followed by a 30‐min incubation with secondary antibodies conjugated to Alexa Fluor 488 (Invitrogen, Life Technologies, Carlsbad, CA). Finally, beads were analysed using fluorescence microscopy.

### 2.8 Cryo‐EM of EVs and actin analysis

Cryo‐EM allows imaging of samples without the addition of any heavy metals or fixatives, which might cause artefacts, with the drawback of yielding a lower contrast. The sample is frozen so rapidly that the water vitrifies forming no ordered crystals, and the native structure of the sample is preserved. Freshly prepared EVs in the 10,000 × *g* pellet, released from 7.5 × 10^7^ astrocytes, were resuspended in saline and vitrified by applying a 3.5 μl droplet onto a holey carbon grids (Copper 300‐mesh Quantifoil R2/1) glow discharged for 45 s at 40 mA using a GloQube system (Quorum Technologies, East Sussex, UK). After 60 s incubation, the grid was plunge‐frozen in liquid ethane using a Vitrobot Mk IV (Thermo Fischer Scientific, Waltham, MA, USA) operating at 4°C and 100% RH. Images of the vitrified specimen were acquired using a Talos Arctica transmission electron microscope (Thermo Fisher Scientific, Waltham, MA, USA) operating at 200 kV and equipped with a Falcon 3EC direct electron detector (Thermo Fischer Scientific, Waltham, MA, USA). Images were acquired with an exposure time of 1 s and a total accumulated dose of 80 electrons per A2 at a nominal magnification of 45,000 ×, corresponding to a pixel size of 2.29 Å/pixel at the specimen level, with applied defocus values between 2 and 4 μm. Images acquired with Volta phase‐plate were recorded at defocus values between 0.5 and 1 μm. Contrast transfer function (CTF) estimation was performed using CTFFIND4 (Rohou & Grigorieff, 2015). All EVs contained within the image were measured using ImageJ software. EVs were classified in single round EVs, oval EVs, tubular EVs, irregular EVs or double/multilamellar EVs. Oval EVs and tubular EVs were measured along their long axis.

Actin filament picking, segments extraction and analyses were performed using RELION‐3 software (Zivanov et al., 2018). Filaments were manually picked using RELION's helix picker and a total of 1,381 segments were extracted using a box size of 100 pixel and inter‐box distance of ∼10% (Rohou & Grigorieff, 2015; Zivanov et al., 2018).

### 2.9 Subcellular localization of large mCLING‐labelled EVs

EVs in the 10,000 x g pellet were resuspended in 500 μl of sterile and 0.1 μm filtered PBS and incubated with 400 nM mCLING‐ATTO 647N‐labeled (Synaptic System, Goettingen, Germany) in a black tube on ice for 5 min. The reaction was quenched by adding 500 μl of 1% BSA in PBS. Then EVs were diluted in 10 ml of PBS, re‐pelleted at 10,000 x g to eliminate the dye excess, resuspended in neuronal medium and added to membrane‐targeted GFP‐transfected hippocampal neurons for 1 h before fixing the cells with 4% paraformaldehyde for 15 min. Coverslips were then mounted on a microscope slide and Z‐stacks were acquired with a Zeiss LSM800 confocal microscopy (Oberkochen, Germany). Analysis of EV localization on neuron processes and cell bodies was developed with ImarisViewer 9.7.0 in the Z‐projection. EVs interacting with thin processes (≤ 2 μm), onto we usually placed EVs by optical manipulation, were distinguished from those localized on primary dendrites in continuity with the cell body.

### 2.10 Optical tweezers (OT)

An IR laser beam (1064 nm, CW) for trapping was collimated into the optical path of an inverted microscope (Axiovert 200 M, Zeiss, Oberkochen, Germany) through the right port of the microscope. The trapping beam was directed to the microscope lens (Zeiss 63X objective, oil immersion, NA 1.4) by the corresponding port mirror (100%) and the tube lens. Optical trapping and manipulation of large EVs was performed following the approach previously described (Prada et al., 2016). Immediately before recording, neurons were washed to remove EVs constitutively released by neurons, and large EVs produced by 2.5 × 10^6^ astrocytes pre‐loaded with Calcein (FilmTracer Calcein Green Biofilm Stain, Life Technologies, Carlsbad, CA, USA; 26 μM for 1 h) were added to neurons plated on glass coverslips and maintained in 400 μl of neuronal medium in a 5% CO_2_ and temperature‐controlled recording chamber at 37°C. Calcein positive large EVs are about 77% of the EVs labelled by mCLING (n = 53) and 72% of EVs detectable in bright field (n = 134). As soon as a Calcein‐positive EV appeared in the recording field (Supplementary Figure 1a), it was trapped and positioned on a selected neuron by moving the cell stage horizontally and the microscope lens axially. EVs larger than 200 nm were trapped and placed in contact with neurons although we cannot exclude that even EVs < 200 nm, due to the diffraction limit, could be imaged as EV with size = 200 nm and monitored in live imaging. Usually 2 EVs per coverslip were recorded. As we learned by practice to recognize exogenous EVs loaded in the medium, entering the recording field, experiments with PrP knock out and FVB wild‐type cultures were carried out with EVs not loaded with calcein. After about 30 s from contact, the laser was switched off to prove EV–neuron interaction. The percentage of EV adhesion was calculated as ratio of EVs that remained attached to neurons after laser switching off over total EVs delivered to neurons by optical manipulation. The percentage of moving EVs was calculated as ratio of EVs showing a displacement from the contact point > of the EV diameter over total adherent EVs. During the experiments neurons were live imaged using a digital camera (High Sensitivity USB 3.0 CMOS Camera 1280 × 1024 Global Shutter Monochrome Sensor, Thorlabs, Newton, NJ, USA) at a frame rate of 2 Hz. To monitor filopodia formation, RFP‐transfected cultures were visualized with a 63× objective using an Axiovert 200 M (Zeiss, Oberkochen, Germany) confocal system equipped with a spinning disk (UltraVIEW acquisition system, Perkin Elmer, Waltham, MA, USA) prior and 10, 20–40 min after the contact with single EVs.

### 2.11 Tracking of single EV

EV position was determined for each video frame (2 frames every 5 s) using a custom MATLAB code (it.mathworks.com). To characterize the EV displacement on the neuron process, 2 distances were calculated: the maximum distance the EV reached from the initial position (in both directions), and the length of the path travelled by the EV in 20 min. Mean velocity and distances were extracted from EV coordinates using a custom R code that exploits the Pythagorean theorem to reconstruct the EV path point‐to‐point (www.r‐project.org). As the code works well only for straight path, the distance from the starting point was calculated using ImageJ software (www.imagej.nih.gov/ij/), when the EV trajectory was not linear, tracing the path manually. The R custom code used for EV analysis is available at https://doi.org/10.6084/m9.figshare.12808211.v1. We classified as ‘static EVs’ i) the EVs with net displacement < of the EV diameter and ii) EVs showing only random (Brownian) motion. 

### 2.12 Fura‐2 videomicroscopy

A total of 14 DIV hippocampal neurons were loaded with 2 μM Fura‐2 (Invitrogen, Carlsbad, CA, USA) pentacetoxy methylester for 45 min at 37 °C, washed and transferred to the recording chamber of an inverted microscope (Axiovert 100; Zeiss, Oberkochen, Germany) equipped with a calcium imaging unit Polychrome V (TILL Photonics GmbH, Planegg, Germany). Images were collected with a CCD Imago‐QE camera (TILL Photonics GmbH, Planegg, Germany) and analysed with FEI Live Acquisition 2.6.0.14 software (FEI GmbH, Munich, Germany). After excitation at 340 and 380 nm wavelengths, the emitted light was acquired at 505 nm at 1 Hz. Calcium concentration was expressed as F340/F380 fluorescence ratio. The ratio values in selected region of interest corresponding to neuronal cell bodies were calculated from sequences of images to obtain temporal analysis.

### 2.13 ATP measurements in EVs

The biological assays for ATP detection were performed modifying previously described methods (Coco et al., 2003; De Marchi et al., 2020), using fura‐2 loaded oligodendrocytes or melanoma B16‐F10 expressing pmeLUC as ATP sensor cells. For the fura‐2 assay, large EVs, derived from 5 × 10^6^ cells under ATP stimulation or constitutively released by 10 × 10^6^ cells were resuspended in 200 μl of KRH and split into two aliquots before testing on oligodendrocytes. Only one aliquot was pretreated with Apyrase (30 units/ml, Sigma, St. Louis, MO, USA) for 15 min to assess whether the calcium responses evoked by the EVs in oligodendrocytes were due to EVs‐associated ATP. The other aliquot was used as a positive control. To damage the EV membrane and favour ATP leakage into KRH, one aliquot of EVs was subjected to 3 cycles of freeze and thaw: for every cycle, EVs (resuspended in 100 μl of KRH) were immersed in liquid nitrogen for 30 s (freeze) and then put in a warm bath at 37°C for 30 s (thaw). To deplete large EVs from their luminal content, EVs were broken or not by hypo‐osmotic stress (via suspension in 500 μl of 7.3 mM PBS at 4°C for 30 min) and re‐pelleted at 100,000 × *g* for 1 h before being resuspended in 100 μl of KRH and tested on oligodendrocytes. For the pmeLUC assay (De Marchi et al., 2019, 2020; Pellegatti et al., 2005), 10 × 10^3^ B16‐F10 pmeLUC cells were seeded into a 96‐wells plate in complete RPMI‐1640 medium (Sigma‐Aldrich), let adhere and grow for 24 h. Subsequently, 15 × 10^6^ astrocytes were incubated with 100 μM BzATP (Sigma‐Aldrich, St. Louis, MO, USA) for 30 min in 7 ml of KRH. Large EVs were collected as previously described and resuspended in sterile PBS (Euroclone, Milan, Italy). D‐Luciferin (Promega, Madison, Wisconsin, USA) was added to the wells containing B16‐F10 pmeLUC cells at a 60 μg/ml concentration. The basal luminescence emission was acquired at medium binning for 10 min with an IVIS Lumina luminometer (Perkin Elmer, Milan, Italy). Cells were then incubated with 10 μl of EV suspension or 500 μM ATP (Sigma‐Aldrich), and luminescence emission was acquired for extra 10 min. Luminescence data were analysed with the Living image Software (Perkin Elmer, Milan, Italy) and expressed as photons/second of acquisition.

### 2.14 Immunocytochemistry

Hippocampal neurons from FVB mice, plated on 18 mm glass coverslips were fixed with 4% paraformaldehyde for 15 min. Samples were then incubated with GSDB blocking solution (Goat Serum Dilution Buffer; containing goat serum, phosphate buffer and sodium chloride) in non‐permeabilizing conditions for 30 min at RT. Coverslips were then incubated with primary mouse antibodies anti‐PrP (W226) and anti‐NCAM (AB5032, Merk Millipore) diluted in GSDB for 1 h, followed by species‐specific fluorochrome‐conjugated secondary antibodies: anti‐Mouse Alexa Fluor 488, anti‐Rabbit Alexa Fluor 594 and DAPI (4′,6‐diamidino‐2‐phenylindole, Invitrogen), to stain nuclei. Coverslips were mounted on microscope slides and images were acquired with a Zeiss LSM800 (Oberkochen, Germany) confocal microscope and processed with ImageJ software.

### 2.15 Data analysis

Statistical analysis was performed on all datasets. If not otherwise specified, data are expressed as means ± SEM. Data were first tested for normal distribution with GraphPad Prism 6 software, then the appropriate statistical test has been used (see figure legends). The accepted level of significance was *P* ≤ 0.05, indicated by one asterisk; those at *P* ≤ 0.01 are indicated by double asterisks, the ones at *P* ≤ 0.001 are indicated by triple asterisks and *P* ≤ 0.0001 are indicated by four asterisks.

## 3 RESULTS

### 3.1 Large EVs produced from primary rat astrocytes have different morphologies

**FIGURE 1:**
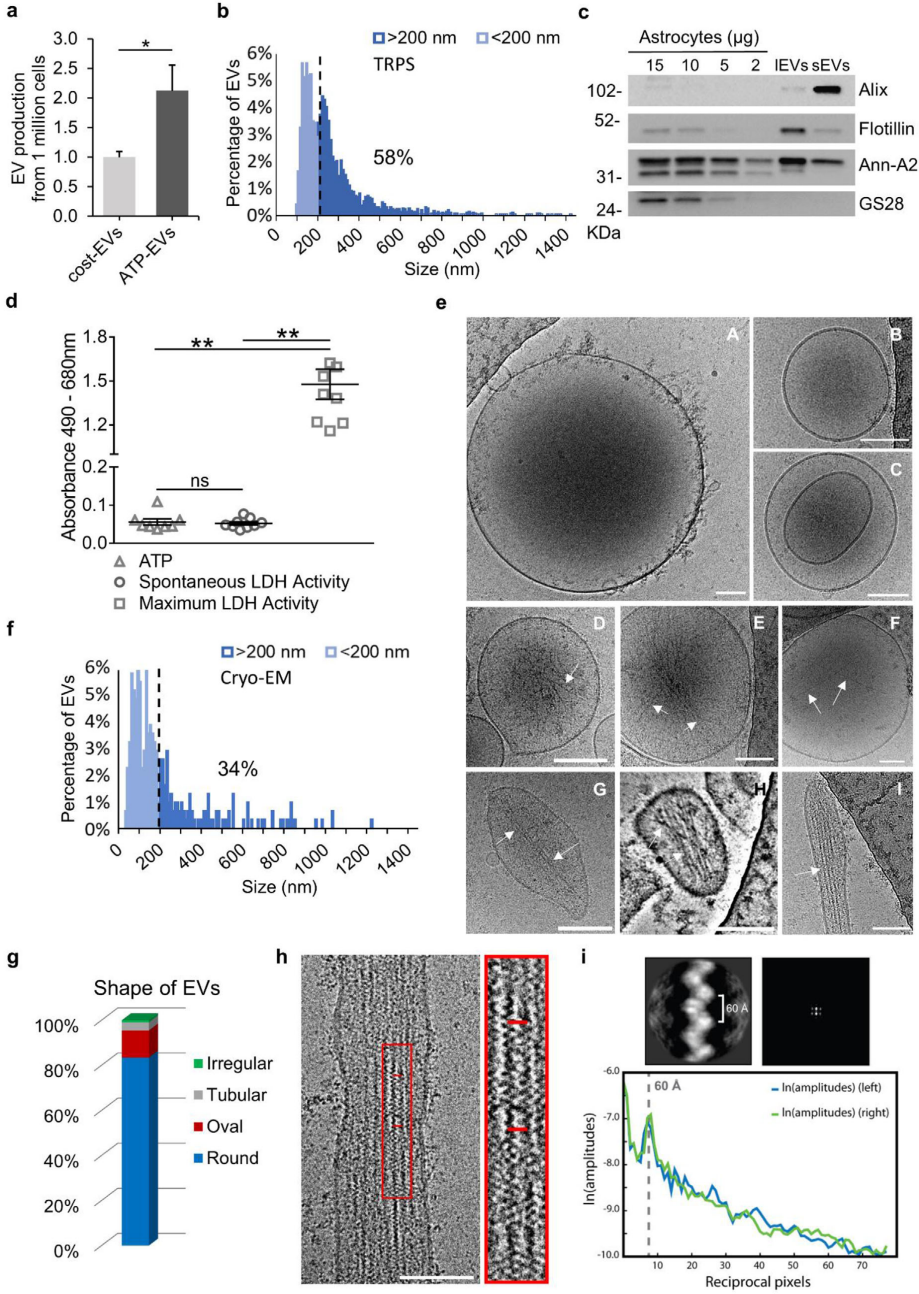
Ultrastructure of medium/large EVs released from ATP‐stimulated astrocytes. (a) Histogram shows basal and ATP‐induced production of EVs in the 10,000 × *g* pellet from cultured astrocytes. The number of ATP‐induced EVs detected by TRPS with a qNano in 100 μl of 0.1 μm‐filtered Krebs‐Ringer solution were normalized to constitutive EVs (ATP‐EVs vs. constitutive EVs t test *P*  = 0.033; N = 3). (b) Size distribution of EVs in the 10,000 × *g* pellet according to TRPS. Note that medium/large EVs (> 200 nm) are ∼58% of EV population (n = 2507). (c) Representative immunoblot of astrocytes (15‐ 2 μg of cell lysate), medium/large EVs (lEVs) and small EVs (sEVs) pelleted at 10,000 × *g* and 100,000 × *g* respectively, released from 20 × 10^6^ cells, for the EV marker Alix, Flotillin 1, Annexin A2 and the negative Golgi marker GS28. (d) Basal and ATP‐induced LDH release in the astrocyte supernatant (one‐way ANOVA *P* = 0.0002, Dunn's multiple comparisons test: ATP vs. Maximum LDH release *P* = 0.0011, Spontaneous vs. Maximum LDH release *P* = 0.0012). Maximum LDH release was measured by exposing astrocytes to the lysis buffer. (e) Representative cryo‐electron microscopy micrographs of round EVs (A‐F), made by the characteristic phospholipid bilayer, as well as of less abundant irregular or tubular EVs (G‐I) in the 10,000 × *g* pellet (low‐pass filtered at 40 Å, except for the first micrograph). Note the presence of filaments (arrows) in elongated/tubular EVs (G‐I) but also in a subpopulation of round EVs (D‐F). Scale bars = 100 nm. (f) Size distribution of astrocyte‐derived EVs, detected by cryo‐EM in the 10,000 × *g* pellet. Note that medium/large EVs (>200 nm) are ∼34% of the EV population. (g) Prevalence of astrocytic EVs with different shape and morphologies in the 10,000 × *g* pellet. (h) Cryo‐electron micrograph showing parallel actin filaments in the lumen of a tubular EV. Red rectangle shows the area zoomed on the right. (i) Reference‐free 2D class average of intravesicular actin filaments (top right, box size 229 Å) is shown together with amplitudes in the frequency (Fourier) space (top left) and helical layer line profile

Primary rat astrocytes were exposed to ATP for 30 min to promote EV shedding and medium/large EVs were isolated by ultracentrifugation at 10,000 × *g* after pre‐clearing of cell supernatants from cells and debris at 300 × *g* ^13^. ATP stimulation for a brief period in extracellular saline ensured higher yield of medium/large EVs (Figure 1a), similar in size to constitutive EVs, as indicated by tunable resistive pulse sensing (TRPS), a technique suitable to analyse highly polydisperse EVs (Figure 1b and Supplementary Figure 1a). TRPS analysis revealed that EVs released under ATP stimulation are highly heterogeneous in size and have a mean diameter of 291.39 ± 3.58 nm (Figure 1b). According to this method, medium/large EVs (> 200 nm) were ∼58% of the whole EV population. Short ATP stimulation also ensured high purity of EV preparation without the need of further purification steps. This was indicated by Western blot analysis of medium/large EVs for positive and negative EV markers compared to cell lysate/small EV fraction, isolated at 100,000 × *g* (Figure 1c). Analysis of lactate dehydrogenase (LDH) revealed no difference between the medium collected at the end of ATP stimulation and under constitutive conditions (Figure 1d), excluding a link of EVs released upon short ATP stimulation to an apoptotic fate of the cells. Morphological analysis using high resolution cryo‐electron microscopy (cryo‐EM) (Yuana et al., 2013; Zabeo et al., 2017) revealed that most EVs in the 10,000 × *g* pellet were rounded in shape (83%, Figure 1e, g) and made by a single lipid bilayer (80%), although very heterogeneous in size (Figure 1e‐f). Their size range was from 20 nm to 1300 nm (mean ± SEM 217 ± 12 nm, Figure 1f; number of EVs n = 307) with large EVs (> 200 nm) being 34% of the whole EV population, a lower percentage compared to TRPS. However, cryo‐EM may underestimate large EV presence in the sample, as large EVs may not be included in the ∼100 nm ice film for cryo‐EM detection. A subpopulation of EVs were multi‐lamellar, i.e. contained two or more vesicles in their lumen (20%, Figure 1e panel C), while a minor percentage of EVs had a tubular (3.6% Figure 1e panel I and Figure 1g) or irregular shape (1%, Figure 1e panel G‐H and Figure 1g). Other peculiar features of some EVs were rough surface (2.6%, Figure 1e panel A), likely due to the presence of transmembrane proteins, electron dense content (24%, Figure 1e panel A, C) and presence of actin filaments in their lumen (2%, Figure 1e panel D‐I), a typical feature of larger EVs from our preparation, with a tubular/elongated shape (mean major axis = 435 ± 70 nm; n = 12), whose frequency may be under underestimated by cryo‐EM. In tubular/elongated EVs actin filaments were oriented in parallel to one another and extended along the tubule (Figure 1e panel H‐I and Figure 1h). Actin filaments in tubular EVs displayed canonical width of ∼7 nm. Inter‐filament distance was ∼10 nm (Figure 1i) with inter‐subunit distance of ∼60 Å (Figure 1i). In round EVs, filaments were randomly distributed, and their structure was not sufficiently defined to resolve their molecular composition (Figure 1e panel D‐F). Cryo‐EM confirmed that EVs were not contaminated with apoptotic bodies, intracellular organelles derived from damaged cells nor protein aggregates (Supplementary Figure 1c).

### 3.2 Single‐particle tracking of astrocytic EVs on the neuron surface

**FIGURE 2:**
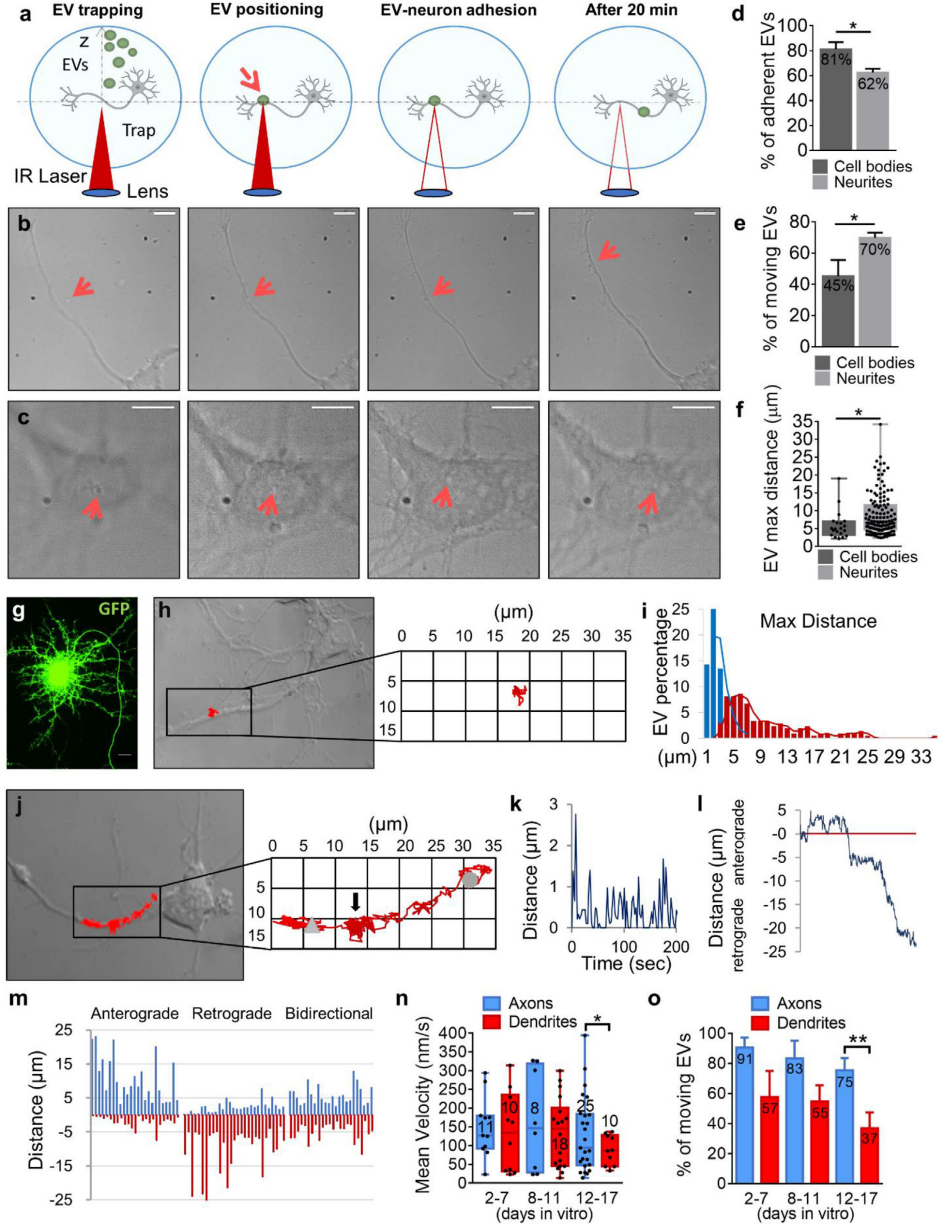
Features of large EV motion on the neuron surface. (a) Schematic representation of single EV delivery to neurons by optical tweezers. EV is first trapped above the neurons by the infrared (IR) laser tweezers (left), then the stage is moved in plane (XY) to reach the target neurons. Then, the trap is moved axially (Z) to set the EV in contact with the neuron (middle). The trapping laser is switched off (middle) and EV‐neuron interaction is then monitored by time lapse imaging up to 20 min (right). (b‐c) Sequence of phase contrast images showing examples of EVs driven to the growing axon (top) or the neuron cell body (bottom) following the procedure described in A (scale bar = 10 μm). (d) Percentage of large EVs that adhered to the neuron cell bodies and processes (cell bodies n = 71, number of independent experiments N = 13; processes n = 396, N = 95, Mann‐Whitney test, *P* = 0.0271). (e) Percentage of large EVs displaying motility on the cell bodies and neurites (cell bodies n = 54, N = 13; processes n = 212, N = 95, Mann‐Whitney test, *P* = 0.0138). (f) Maximal distances from the contact point reached by all EVs in 20 min (cell bodies n = 20, processes n = 135, Mann‐Whitney test, *P* = 0.0118). (g) Representative fluorescence image of a neuron transfected with GFP to delineate dendrite and axon morphology. Scale bar = 10 μm. (h,j) The trajectory of EVs are represented in red, superposed on the phase contrast images of developing neurons and enlarged in the insets. The EV in h shows radial motion while the EV in j exhibits directional motion towards the cell body. Grey arrowhead indicates the starting point, grey circle the ending point. (i) Distribution of maximal distances from the contact point of EVs delivered to neurites (n = 209). Static EVs are in blue, moving EVs are in red. (k) Distances travelled by the EV in j for 200 s after adhesion and sampled every 2.5 s, illustrating stop‐and‐go movement. (l) Temporal plot of the distance from the contact point of the EV in j during 20 min of recording sampling at 2 Hz, showing both anterograde and retrograde motion. (m) Distribution of maximal positive and negative distances from the contact point of EVs moving along neurites of both young and differentiated neurons (n = 84), showing anterograde (31%), retrograde (35%) or bidirectional (35%) motion. EVs were delivered to neurons. (n) Velocity of EVs (nm/sec) on dendrites and axons at different stages of neuronal development (12‐17 DIV, one‐tailed unpaired t test with Welch's correction, *P* = 0.0450 axons vs. dendrites). Number of EVs are indicated in the bars. (o) Movement of large EVs on dendrites and axons at different stages of neuronal development (12‐17 DIV, Mann‐Whitney test, *P* = 0.0075 axons vs. dendrites). Percentage of moving EVs are indicated in the bars (EVs on axon, n = 33; EVs on dendrites n = 41)

To elucidate whether astrocytic EVs in the 10,000 × *g* pellets interact with neurons at preferential sites, we gently placed single EVs on different compartments of hippocampal neurons (cell body, dendrite and axon) by using optical manipulation (Prada et al., 2018) and examined EV‐neuron interaction through live microscopy, starting from the observation that EVs loaded in the cell medium can randomly attach both to the soma and the neurites. EVs in the 10,000 *x* *g* pellet were added to neuronal medium, trapped by infrared laser tweezers and positioned on the selected compartment. Thirty seconds after contact the trapping laser was switched off and time‐lapse images were collected at 2–20 Hz for 20 min to monitor EV‐neuron dynamics (Figure 2a). Medium/large EVs (> 200 nm, accounting for about 60% of the 10,000 × *g* EV population according to TRPS analysis) were mostly imaged, as small EVs may be too tiny to be visualized in light microscopy and delivered to neurons. We found that this population of EVs, for simplicity called large EVs from now on, adhered more efficiently to the cell body (81% of adhesion) than neurites (62% of adhesion) (Figure 2b‐d, Movie S1 and Movie S2 online with this article). This difference might be due to differential expression of EV interacting molecules on the two neuronal compartments or the fact that the soma offers a larger surface around EV contact point, making easier for the EV to find their specific surface interactors and/or undergo an higher number of interactions. However, we cannot exclude that higher adhesion of large EVs to the cell body might also result from better accuracy in placing the EVs on a larger cell surface. About 50% of adherent large EVs were round in shape, while the remaining were tubular (9%) or oval/irregular (41%), as analysed after turning the infrared laser off, to avoid possible EV distortions (Supplementary Figure 2, n = 212).

Surprisingly, after adhesion a large fraction of EVs (58% ± 3.25%, n = 266, number of independent experiments N = 107) displayed net movement from the contact site, surfing on the plasma membrane of neurons. Although phase contrast resolution did not always allow to discriminate between intra‐ and extra‐cellular EV movement, EV recapturing by the laser tweezers in close proximity to contact site of the EV on neuron blocked 100% of EV motion (Table 1, n = 25/25) (Movie S3) while didn't hamper the transport of intracellular organelles (Movie S3), indicating that EVs were moving outside neurons. In addition, some EVs (n = 11/212) jumped from one process to another (Movie S4), even belonging to different neurons, allowing neuron‐to‐neuron EV transition (Movie S5), confirming extracellular EV motion. A total of 70% ± 3.34% of EVs were in motion along neurites (n = 212, N = 95) (Figure 2b, e, Movie S1) and run sizable length (mean length = 143 ± 9.95 μm/20 min) to a maximal distance of 9 ± 0.51 μm from the contact point (Figure 2f). A lower percentage of large EVs moved on the cell body (44 ± 10.50%, n = 54, N = 13) (Figure 2e). Their motion was restricted to the cell body, with no EVs passing from cell body to neurites (Figure 2c, Movie S2). After placement on the cell body, large moving EVs could be recaptured by the laser trap (Table 1, n = 10/10), proving that they were outside neurons, whereas only 27% of ‘static’ EVs, that remained adherent to the place of delivery, were recaptured by optical tweezers, being transiently attracted towards the laser (Table 1, n = 4/15). Although we could not exclude that the laser trapping force (maximum 50 piconewton for our setup) might not be able to re‐trap EVs strongly adhering to the neuron surface (Ashkin, 1992; Bernecker et al., 2021), this suggested that a fraction of large EVs may undergo internalization into the neuron cell body. To assess this hypothesis, we labelled large EVs with the fluorescent dye mCLING (Brenna et al., 2020) and analysed by confocal microscopy the localization of mCLING‐labelled large EVs 1 h after in bulk addition to neurons transfected with membrane‐targeted GFP. This analysis confirmed that a major fraction of large EVs (94%, n = 33) are taken up by neurons in the cell bodies (Supplementary Figure 3).

**TABLE 1:** Recapturing by the laser tweezer of moving and not moving large EVs positioned on processes or cell bodies of hippocampal neurons

	Recapturing of large EVs					
	Moving EVs			NOT moving EVs		
Compartment of Adhesion	Recaptured	Total	%	Recaptured	Total	%
Neurites	15	15	100	12	24	50
Cell Bodies	10	10	100	4	15	27
Total	25	25	100	16	39	41

Run length (102 ± 22.37 μm/20 min) and maximal distance from the contact point (6 ± 0.88 μm, Figure 2f) of large EVs moving on the cell bodies were shorter compared to neurites (run length soma vs. neurites, Mann‐Whitney test, *P* = 0.030). Thus, EVs moving on neurites, either dendrites or axons, were selected for further analysis. Axons were distinguished from dendrites by their longer length and their smaller size in developing cultures (≤12 days in vitro), and by the absence of spines in mature cultures transfected with GFP (≥ 13 days in vitro) (Figure 2g).

### 3.3 Directional movement of large EVs along neuronal processes

By a custom MATLAB code, we analysed the dynamics of all large EVs which adhered to neurites, including those that remained anchored to the contact site. Among the latter (∼35% of total EVs) only few EVs were virtually immobile (EV displacement < EV diameter) while the remaining fraction showed random Brownian motion, restrained by a tether to the neuron membrane (Figure 2h). The mean radial displacement from the attachment point was 1.8 ± 1.1 μm. For simplicity, both virtually immobile EVs and EVs displaying only radial motion are referred to as ‘static EVs’ hereafter (Figure 2i blue bars).

Most large EVs (n = 135/209) exhibited both radial and directional movement (mobile EVs, Figure 2i, red bars), with a prevalent directional component (Figure 2j). Directional movement was typically intermittent, characterized by interruptions between periods of motion (‘stop and go’ motion). ‘Stop’ intervals are indicated by 0 values in the temporal plot of distance travelled by EVs in 200 s (Figure 2k). On average, EVs were in motion for nearly half the recording time (45% ± 2.2%, n = 209). Radial motion was evident during ‘stop’ intervals (Figure 2j arrow) or at high frequency (20 Hz) acquisition (Movie S6). A typical feature of directional movement was back and forth motion, highlighted in the temporal plot of EV distance changes from the contact point, showing both positive and negative variations (Figure 2l). As a consequence of back and forth movement, large EVs repeatedly localized at the same sites on axons, dendrites or their protrusions (filopodia/dendritic spines). Movie S1 shows the typical trajectory of an EV that retraced several times the same regions of a growing axon while exploring filopodia on the way to the growth cone. Visualization of EV trajectories in neurons cultured in vitro for 2–17 days revealed that most EVs moved along axons and dendrites in a prevalent direction (run length > 2 × length in the opposite direction), either retrograde, towards the neuron cell body, or anterograde, away from the soma. However, a significant fraction of EVs (n = 29/84) were characterized by truly bidirectional movement, running similar length along neurites in both directions, as indicated by the plot of the maximal positive and negative distances from the contact point (Figure 2m). Importantly, along mature axons half of large EVs (50%) moved in an anterograde direction (n = 14, N = 9). The speed of large EVs along neurites ranged from 0.011 to 0.412 μm/sec (mean speed = 0.126 ± 0.008 μm/sec, n = 135). EV velocity was similar on axons and dendrites in immature neurons, up to 11 days in vitro (Figure 2n). With age, and in parallel to increased complexity of dendrites in terms of morphology and composition, both EV speed (Figure 2n) and the percentage of moving EVs decreased (Figure 2o) on dendrites. Conversely, EV speed did not change on axons, with a significant difference between the two processes in neurons ≥12 days in vitro (Figure 2n).

### 3.4 Stable contact of large EVs at the neuron surface promotes formation of novel protrusions

Next we asked what happens at preferential contact points, where large EVs stop moving and at places of stable adhesion, where EVs immediately establish a stable interaction with neurites. We found that 50% of large EVs (n = 12/24) could be recaptured at sites of persistent contact on neurites, as indicated by transient shift of EVs towards the laser trap (Table 1, Supplementary Figure 4). This suggested that at least half large EVs were still outside neurites at the end of recording (20 min). Further analysis of mCLING‐labelled large EV localization in neurons transfected with membrane GFP showed that the vast majority of EVs (94% n = 69) was still outside thin (≤ 2 μm) neurites 1 h after EV addition (Supplementary Figure 3), while along primary dendrites most EVs were internalized (82%, n = 38). Collectively, these data point to large EV size/neurite thickness ratio as key factor retaining EVs at the neuron surface. They also suggest that not all EVs outside neurons can be re‐captured by the laser trap.

**FIGURE 3:**
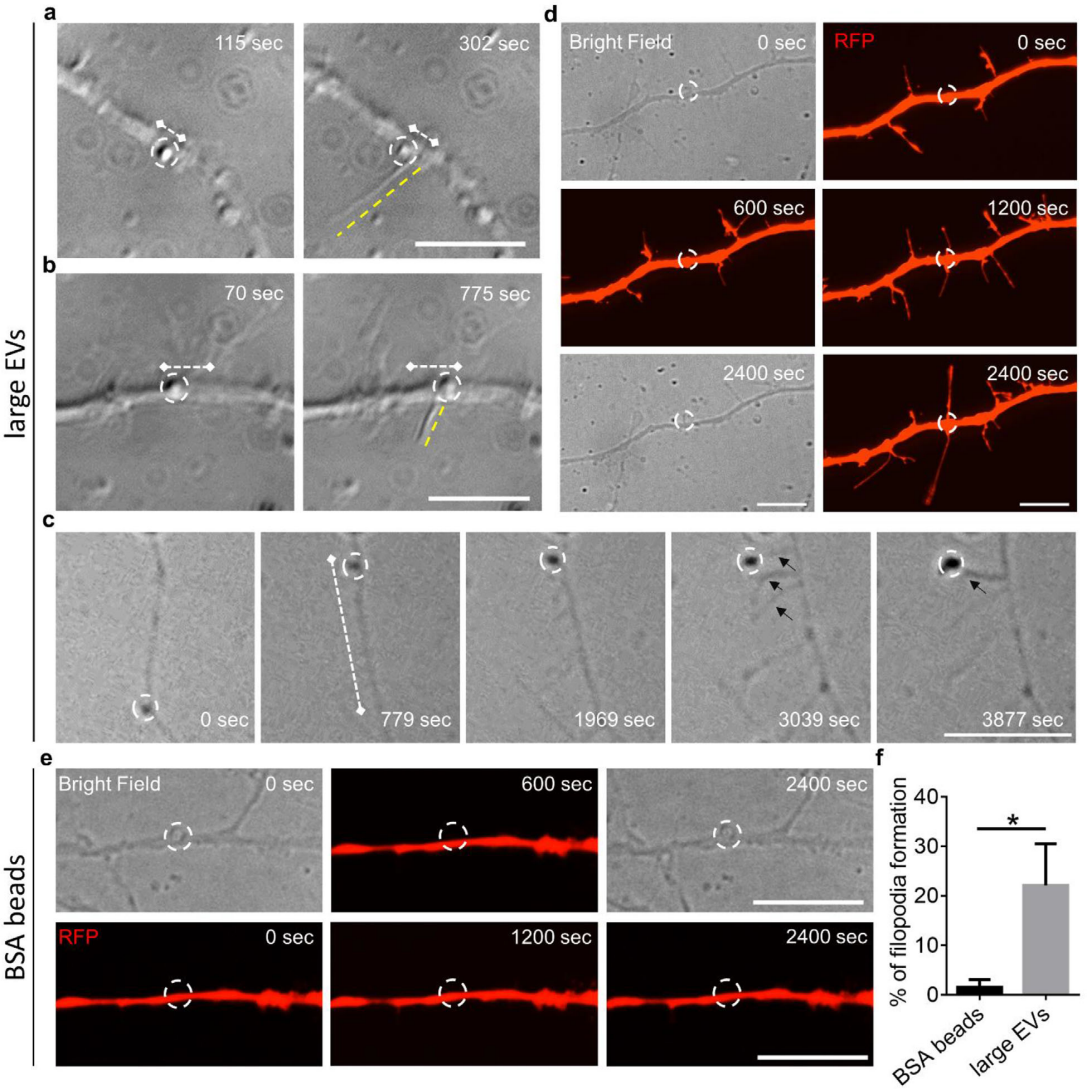
Filopodia formation after persistent contact of large EVs with neuronal processes. (a‐b) Phase contrast images showing formation of filopodia (yellow dotted line) at the stop site of large EVs (white dotted circle). White dotted lines indicate EV displacement. (c) Phase contrast images of a fast moving large EV (white dotted circle) that induced formation of multiple filopodia (black arrows) around the stop site on the neurite. White dotted lines indicate EV displacement. (d) Phase contrast (bright field) and fluorescent confocal (RFP) images showing formation of two opposite protrusions at the large EV‐neuron contact site (white dotted circle), 1200 s after adhesion. The EV is out of focus in bright field; the focus plane is on the underlying dendrite. (e) Representative images showing the morphology of a neurite placed in contact with a BSA‐coated bead (white dotted circle) at the indicated time points after adhesion. Scale bars = 10 μm. (f) Quantification of new filopodia formation following adhesion of large EVs or BSA‐coated beads (BSA beads) to RFP‐transfected neurites (BSAb n = 38, N = 5 vs. EVs n = 30, N = 5, Mann‐Whitney test *P* = 0.0476)

Interestingly, time‐lapse imaging revealed that astrocyte‐derived EVs have the ability to induce formation of new protrusions at or around the sites of persistent contact (Figure 3a‐c, n = 9/32 N = 9). Protrusions formed after 3–20 min of persistent contact. Novel protrusions were not always detectable in bright field while were easily visible by confocal microscopy, which allowed to capture thin filopodia budding from RFP transfected dendrites (Figure 3d, n = 6/30 N = 12). In one particularly striking case (Figure 3c), a moving EV induced formation of multiple filopodia around the stop site on the dendritic shaft. One filopodium budded exactly from the contact site and grew under the EV. Other filopodia appeared around the stop site and then converged towards the EV, ultimately leading to a single branch/protrusion. Carboxylated microsynthetic particles, covalently coupled with BSA, were used as negative control for filopodia formation driven by astrocyte large EVs. New filopodia were very rarely observed upon delivery of BSA‐coated beads (BSAb) in bright field (BSAb n = 2/42 N = 8 vs. EVs n = 9/32 N = 9, t test *P* = 0.0017) and by confocal microscopy (BSAb n = 1/38 N = 5 vs. EVs 6/30 N = 5) (Figure 3e‐f), ensuring the specificity of astrocytes‐derived EVs in inducing new filopodia. Taken together these findings suggest that large astrocytic EVs may scan the neuron surface in search for specific spots of the plasma membrane where new protrusions can be induced.

### 3.5 Motion of EVs depends on intact actin cytoskeleton in neurons

**FIGURE 4:**
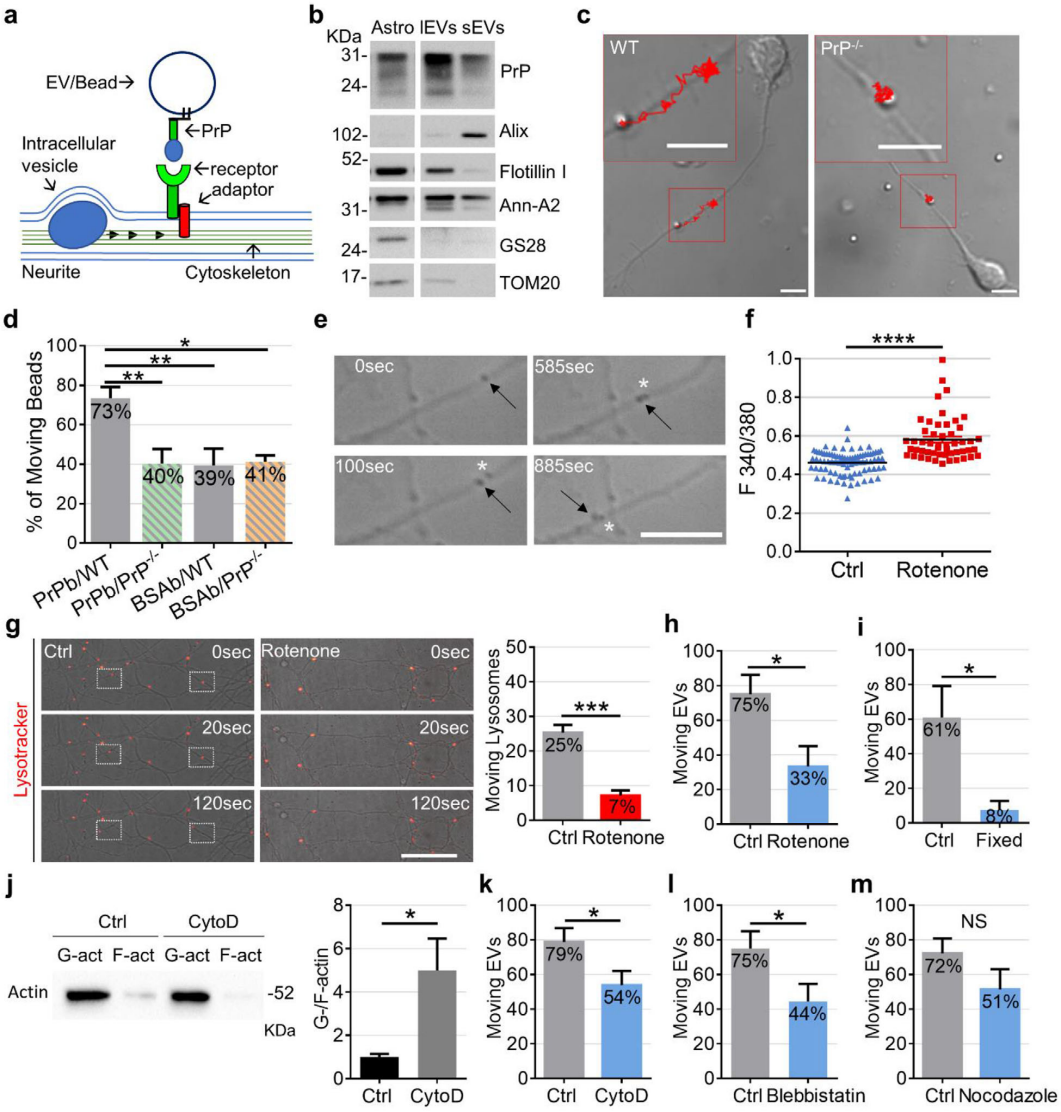
Actin cytoskeleton dynamics drive extracellular motion of large EVs. (a) The scheme shows possible coupling of EV to the moving cytoskeleton of neuron via binding to a neuronal receptor. It also shows that transport of large intracellular vesicles along the cytoskeleton may alter extracellular EV motion by dragging the cytosolic portion of EV receptor or adaptor protein. (b) Immunoblot of astrocytes (at decreasing loading amounts), large EVs (lEVs) and small EVs (sEVs) pelleted at 10,000 *x g* and 100,000 × *g* respectively from 20 × 10^6^ cells for PrP, the EV markers alix, flotillin 1 and annexin A2 (Ann‐A2) and the Golgi and mitochondrial markers GS28 and TOM20. (c) Phase contrast images showing the transport of PrP‐coated synthetic particles on the surface of wild type (WT, left) but not PrP^–/–^ (right) growing axons (scale bar = 5 μm) in cultures established from FVB mice (WT and PrP^–/–^). The trajectories of the PrP‐coated beads are represented in red, superposed on the neuron images and shown at high magnifications in the insets. (d) Synthetic beads were coated with full length PrP or C‐terminal form of PrP, control beads were coated with albumin (BSAb). Histograms show the percentage of moving PrP‐coated beads (PrPb) and BSAb on the growing axons of WT and PrP^–/–^ neurons (PrPb on WT neurons (PrPb/WT) n = 26, N = 7 independent experiments, C‐terminal form of PrP n = 13/26; PrPb on PrP^–/–^neurons (PrPb/PrP^–/–^) n = 40, N = 9, C‐terminal form of PrP n = 30/40; BSAb on WT neurons (BSAb/WT) n = 42, N = 8; BSAb on PrP^–/–^neurons (BSAb/PrP^–/–^) n = 39, N = 7; one‐way ANOVA *P* = 0.0047, Turkey's multiple comparison test: PrPb/WT vs. PrPb/PrP^–/–^ *P* = 0.0099, PrPb/WT vs. BSAb/WT *P* = 0.0097, PrPb/WT vs. BSAb/PrP^–/‐^ *P* = 0.0198). (e) Sequence of phase contrast images showing simultaneous motion of extracellular (black arrow) and intracellular (white star) vesicles along a thin process (from movie S7). (f) Control and Rotenone‐treated neurons were loaded with the ratiometric calcium dye FURA‐2 prior to single cell calcium imaging. The values are mean 340/380 fluorescent ratios, a measure of cytosolic calcium concentrations. Dead neurons (340/380 Ratio values > 1) were excluded from the analysis (Ctrl n = 83; Rotenone n = 51; N = 3; Mann‐Whitney test, *P* < 0.0001). (g) Control and Rotenone‐treated neurons were loaded with LysoTracker and the trafficking of LysoTracker‐positive organelles was analyzed by confocal video recording. ROIs indicate moving lysosomes in the overlay of phase contrast with fluorescence images. The percentage of moving lysosomes in control and Rotenone‐treated neurons is shown on the right (Ctrl n = 445; Rotenone n = 284; Mann‐Whitney test, *P* = 0.0010, N = 5). (h) Percentage of large EVs moving along the surface of neurites in control and Rotenone‐treated neurons. Only EVs delivered to neurites of Rotenone‐treated neurons, where lysosome trafficking was blocked, were included in the analysis (Ctrl n = 17; Rotenone n = 24 N = 10, unpaired t test *P* = 0.0206). (i) Percentage of moving EVs on live and fixed neurons (live neuron n = 14 N = 7; fixed neuron n = 28 N = 6; Mann‐Whitney test *P* = 0.0291). (j) Immunoblot of hippocampal neurons extracts for actin showing actin depolymerisation upon CytoD treatment (top). Bottom histogram shows the ratio between G‐actin and F‐actin in control and CytoD‐treated neurons (N = 3, Mann Whitney test, p = 0.05). (k‐m) Percentage of moving EVs on the surface of CytoD‐treated neurons (k; Ctrl n = 21; CytoD n = 33; N = 13; unpaired t test *P* = 0.0390), Blebbistatin‐treated neurons (l; Ctrl n = 23; Blebbistatin n = 27; N = 12; unpaired t test *P* = 0.0426) or Nocodazolo‐treated neurons (m; Ctrl n = 25; Nocodazole n = 21; N = 12; unpaired t test, *P* = 0.1536)

Mechanistically, binding to a neuronal receptor coupled to a moving cytoskeletal network could drive EV motion (Figure 4a). This hypothesis was suggested by two independent observations. First, we found that carboxylated microsynthetic particles covalently coupled with prion protein (PrP), a GPI anchor protein highly enriched in brain EVs (Brenna et al., 2020; Faure et al., 2006) (Figure 4b, Fevrier et al., 2004), were more efficiently transported along neurites compared to control beads, covalently coupled with albumin, when delivered to neurons by optical tweezers, mimicking the trafficking of astrocytic EVs outside neurons (Figure 4c‐d). This observation suggested that binding of PrP to a neuronal receptor enhances passive movement of the synthetic particle on the neuronal surface. Interestingly, a lower percentage of PrP‐coated beads, similar to control beads, were transported along the surface of PrP knock out (PrP^–/–^) neurites (Figure 4c‐d), suggesting that neuronal PrP may act as receptor for PrP coupled to the synthetic beads. Indeed, PrP is capable to undergo homophilic interaction with a PrP molecule in trans and can elicit contact formation (Amin et al., 2016; Malaga‐Trillo & Sempou, 2009; Nguyen et al., 2019). No differences were observed in the fraction of PrP‐coated beads and control beads adhering to either wild‐type or PrP^–/–^ neurites (adhesion of PrP‐coated beads on wild‐type neurons 100%, N = 7; PrP‐coated beads on PrP^–/–^ neurons 100%, N = 9; BSA‐coated beads on wild‐type neurons 79%, N = 8; BSA‐coated beads on PrP^–/–^ neurons 76%, N = 7), excluding a major contribution of neuronal PrP to EV‐neuron interaction.

Second, we found examples of EVs placed on very thin neuronal processes which were induced to move on the neuron surface by the transit of large intracellular vesicles, actively transported along the neuron processes (Figure 4e; Movie S7 and S8; n = 5/135). These observations suggested that the large intraneuronal vesicles, which exceeded the diameter of the neurites, may occasionally drag the cytosolic portion of the EV receptor during their transport along the cytoskeleton, generating simultaneous intra‐ and extra‐cellular vesicle motion for a limited time period (Figure 4e). Despite exploring what type of intracellular organelles may transiently influence extracellular EV motion goes beyond the aim of this study, concomitant monitoring of EVs and LysoTracker red‐labelled vesicles, revealed that EVs often stalled at the same regions of neurites where lysosomes stop, further supporting a link between extra and intracellular vesicle motion (Movie S9).

To assess whether energy‐dependent cytoskeleton rearrangements could drive EV motion on the neuron surface, we first intoxicated neurons with the metabolic inhibitor Rotenone (2μM) for 1 h. Rotenone‐treated neurons exhibited increased cytosolic calcium concentration, revealed by increased F340/F380 fluorescent ratio in cultures loaded with the calcium dye Fura‐2 (Figure 4f), indicating an impairment of plasma membrane ATPase activity. In addition, Rotenone‐treated neurons displayed reduced energy‐dependent trafficking of LysoTracker labelled lysosomes inside the cells (Figure 4g), confirming depletion of energy reserves. Importantly, only 33% of EVs were in motion on intoxicated neurites, where lysosome transport was blocked, while 75% of EVs drifted on the surface of control neurons (Figure 4h). In line with this evidence, acute application of the mitochondria inhibitor Sodium Azide (20 mM) (Svitkina et al., 1986) blocked the movement of a few EVs (n = 3) along neurites (Supplementary Figure 5, Movie S10). To corroborate these findings, we mildly fixed neurons with cold methanol (at 4°C) and placed single EVs on the surface of dead neurons. Only 8% of EVs exhibited a net displacement from the contact point at the surface of dead cells (Figure 4i). Altogether these findings showed that EV motion largely depends on neuron energy metabolism.

To assess the involvement of the cytoskeleton in extracellular EV motion we next blocked actin filaments, a component of the cytoskeleton involved in various types of cellular motility (Rodriguez et al., 2003), by exposing neurons to 1 μM cytochalasin D (CytoD) for 60 min, a drug that binds to actin fast growing end, or to 100 μM Blebbistatin for 30 min, an inhibitor of motor dependent restructuring of actin network (Straight et al., 2003). CytoD‐dependent depolymerisation of actin filament was indicated by increased ratio of globular (G‐) versus filamentous (F‐) actin, as revealed by Western blot analysis (Figure 4j, N = 3). Importantly, by positioning EVs on CytoD‐treated neurites (CytoD) we found no changes in EV adhesion (48% Ctrl n = 57; 47% CytoD n = 79) but a decrease of EV extracellular motion (Figure 4k). Inhibition of actin filaments rearrangements by Blebbistatin caused a significant reduction of EV motion too (Figure 4l), confirming the involvement of the actin cytoskeleton (Supplementary Figure 6). By contrast, depolymerisation of the microtubule cytoskeleton by Nocodazole (10‐30 μM for 60 min), did not alter EV motility (Figure 4m), despite a trend to decrease of EV motion.

### 3.6 Involvement of PrP in EV motion

Drift of astrocytic EVs on neuronal surface strikingly resembles movement of adenoviral particles on target cells, which enhances infection by transporting viruses to sites competent for endocytosis (Ewers et al., 2005;  Lehmann et al., 2005; Schelhaas et al., 2008). Indeed, a parallel between virus and EV extracellular motion has been recently established by a study showing that EV surfing along filopodia facilitates cell entry similarly to virus infection (Heusermann et al., 2016). Binding of viral particles to plasma membrane coxsackievirus adenovirus receptor (CAR), a GPI anchor‐protein, was shown to give rise to extracellular virus motion, by coupling the viruses with the actin cytoskeleton of target cells (Burckhardt et al., 2011). Among surface neuronal receptors which may elicit motion of large glial EVs, our experiments with PrP‐coated synthetic particles (Figure 4c‐d) pointed to the GPI anchor‐protein PrP, as putative linker between EV and the moving actin cytoskeleton. The protein is expressed in astrocytes, in addition to neurons, and highly enriched in EVs thereof (Guitart et al., 2016), especially in large EVs, as indicated by Western blot analysis (Figure 4b).

**FIGURE 5:**
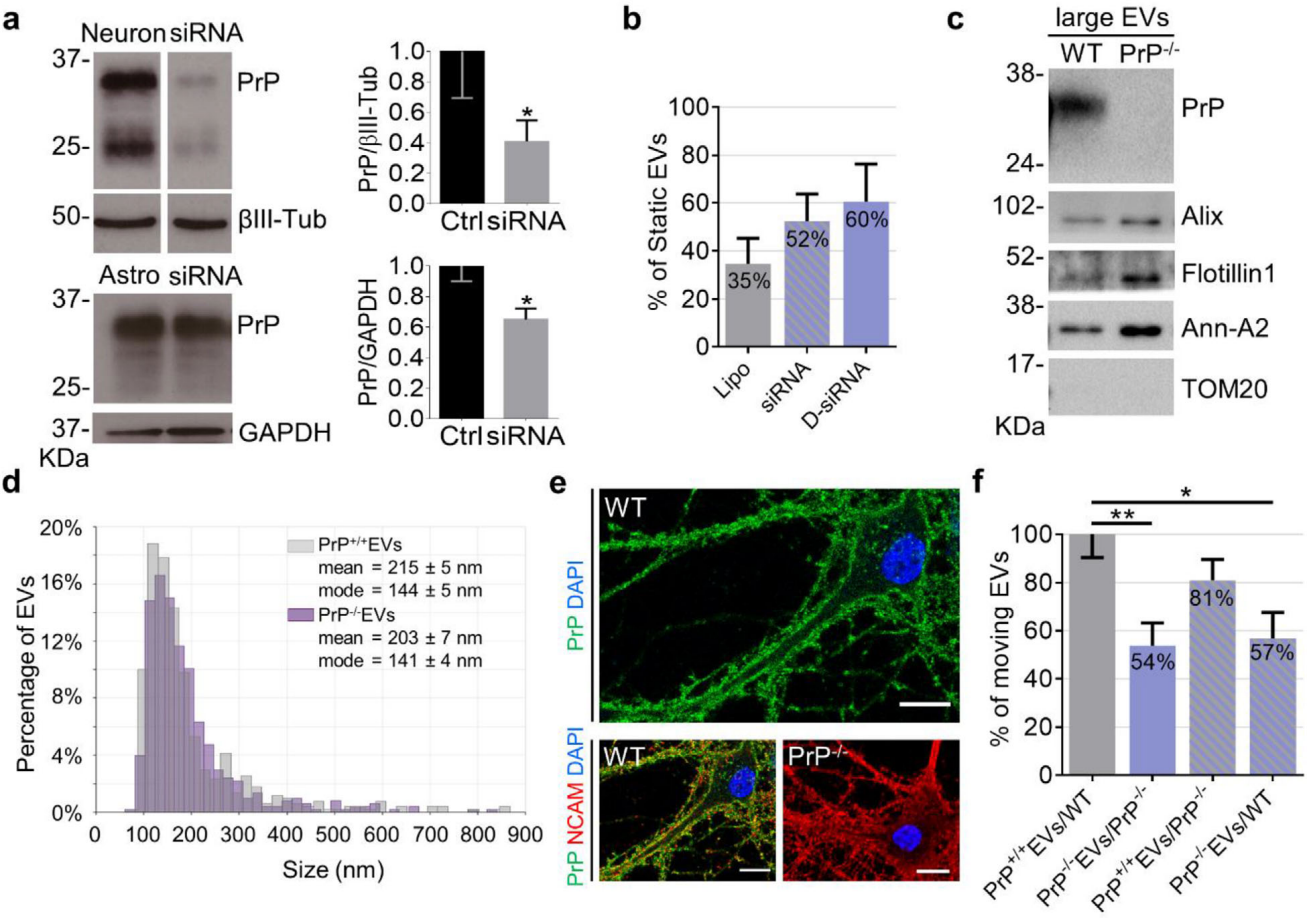
Role of PrP in extracellular EV motion. (a) PrP expression and quantification relative to reference proteins in control and siRNA‐treated neurons (top) and astrocytes (bottom) (neuron N = 7, Mann‐Whitney test one‐tailed *P* = 0.0364; astrocytes N = 3, Mann‐Whitney test one‐tailed *P* = 0.05). (b) Percentage of static EVs from control astrocytes on the surface of lipofectamine‐treated neurons (lipofectamine, Lipo n = 18, N = 5) or PrP‐silenced neurons (siRNA, n = 25, N = 8) and from PrP‐silenced astrocytes on the surface of PrP‐silenced neurons (double siRNA, D‐siRNA, n = 14, N = 4) Kruskal‐Wallis test *P* = 0.4815 and Dunn's multiple comparisons test (not significant). (c) Representative immunoblot for PrP, EV markers and the mitochondrial marker TOM20 of medium/large EVs (l‐EVs) from wild type (WT) and PrP^–/‐^ astrocytes. (d) Representative size distribution of EVs from WT (PrP^+/+^EVs) and PrP^–/–^ astrocytes (PrP^–/–^EVs) (top). (PrP^–/–^EVs vs. PrP^+/+^EVs unpaired t test *P* = 0.0074; N = 3). (e) Representative confocal images of WT and PrP^–/–^ neurons stained for PrP (green), N‐CAM (red) and the nuclear marker DAPI (blue) in non‐permeabilizing conditions. Note that PrP shows a punctate pattern and appears equally distributed in the plasma membrane of neurites and the cell body. Scale bar = 10 μm. (f) Percentage of moving EVs from WT astrocytes on WT neurons (PrP^+/+^EVs/WT, n = 107, N = 17), from PrP^–/–^ astrocytes on PrP^–/–^ neurons (PrP^–/–^EVs/PrP^–/–^, n = 117, N = 14), from WT astrocytes on PrP^–/‐^ neurons (PrP^+/+^EVs/PrP^–/–^, n = 33, N = 10) and from PrP^–/–^ astrocytes on WT neurons (PrP^–/–^EVs/WT, n = 33, N = 8); One‐way ANOVA *P* = 0.0028, Holm‐Sidak's multiple comparisons test: PrP^+/+^EVs/WT vs. PrP^–/–^EVs/PrP^–/–^ *P* = 0.0039, PrP^+/+^EVs/WT vs. PrP^–/–^EVs/WT *P* = 0.0290. Data have been normalized to PrP^+/+^EVs/WT condition

To explore the involvement of PrP in astrocytic EV trafficking along the neuron surface, we first silenced PrP in both neurons and donor astrocytes using specific smart pool siRNAs. Western blot analysis showed that PrP was downregulated by 60% and 40% in neurons and astrocytes respectively, compared to vehicle‐treated cultures (Figure 5a). By positioning single large EVs produced by PrP‐silenced astrocytes onto the processes of PrP‐silenced neurons, we found an increase in the percentage of static EVs (Figure 5b, D‐siRNA), suggesting the involvement of vesicular and/or neuronal PrP in the extracellular motion. Similar results were obtained with control EVs delivered to PrP‐silenced neurons (Figure 5b, siRNA). To corroborate these findings, we isolated large EVs from PrP knock out (Leshchyns'ka & Sytnyk, 2016) astrocytes (PrP^–/–^‐EVs) (Figure 5c). TRPS analysis revealed no significant changes in the average diameter of PrP^–/–^‐EVs in the 10,000 × *g* pellet compared to wild‐type EVs (PrP^+/+^‐EVs) nor in the mode of the diameter (Figure 5d; N = 3). By placing single PrP^–/–^‐EVs on neurites of PrP knock out neurons (PrP^–/‐^ neurons) (Figure 5e‐f) we didn't observe significant changes in EV‐neuron adhesion (67% of adhesion of PrP^–/–^‐EVs on PrP^–/–^ neurons compared to 76% in wild type conditions), excluding that homophilic PrP interaction is necessary for EV‐neuron contact. However, this caused a significant decrease in the percentage of moving EVs (Figure 5f), confirming the involvement of neuronal and/or vesicular PrP in extracellular EV motion. To specifically address the involvement of homophilic PrP interaction in extracellular EV motion we next delivered large PrP^+/+^‐EVs on PrP^–/‐^ neurons and large PrP^–/–^‐EVs on WT neurons. Lack of PrP in either EVs or neurons decreased the percentage of EVs in motion, albeit the difference was significant only upon removal of vesicular PrP (Figure 5f, Supplementary Figure 7). These results suggest that vesicular PrP controls EV transport at the neuron surface by interacting with either PrP, in agreement with PrP‐coated beads data, and/or other PrP binding proteins on neurons.

### 3.7 A fraction of EVs actively moves on neuronal surface

**FIGURE 6:**
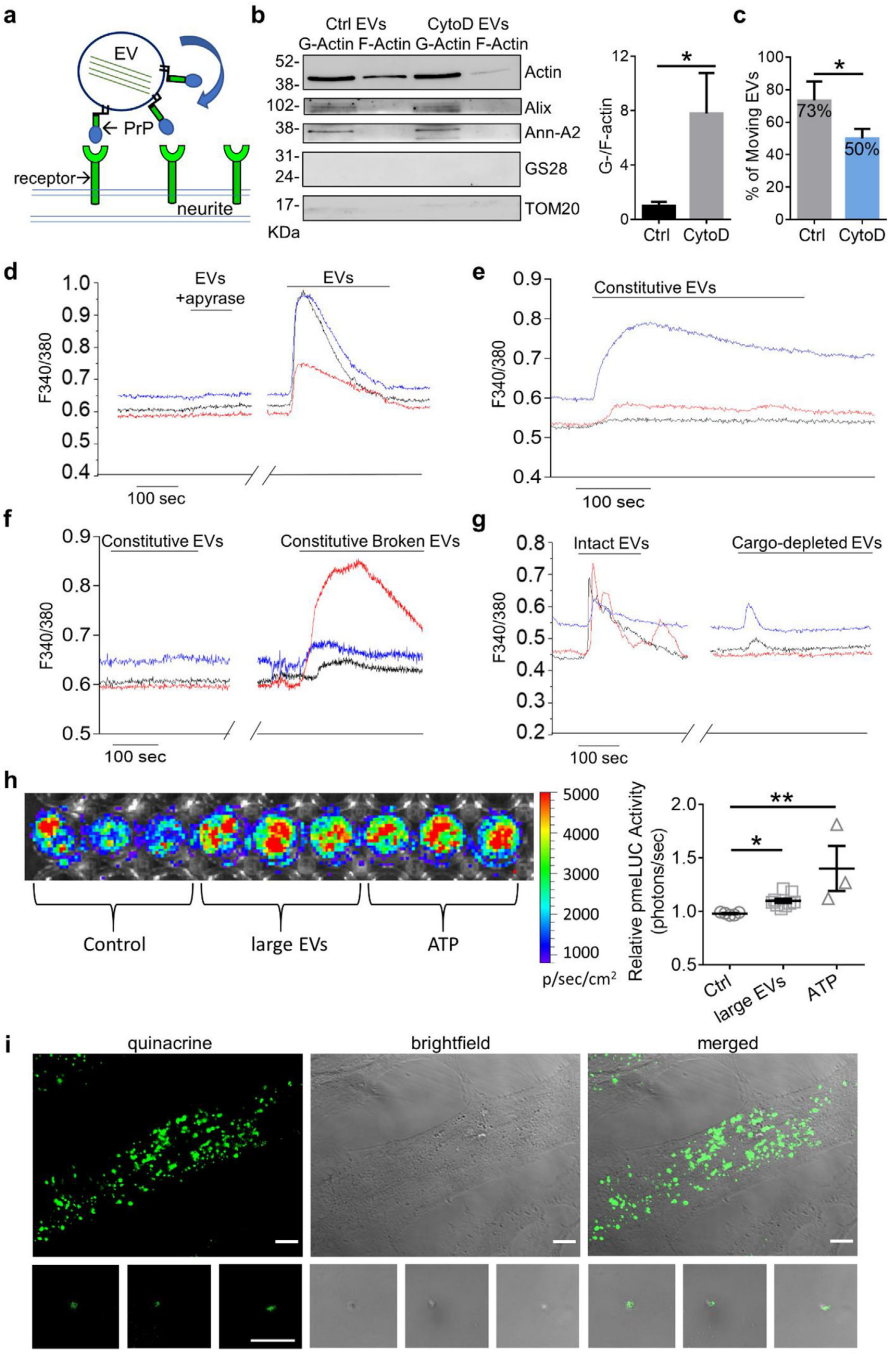
Active motion of astrocytes‐derived EVs. (a) Schematic representation of active motion of EVs, through intermittent contact with neuronal surface receptors. (b) Representative Western blot (left) of F‐actin and G‐actin in control and CytoD‐treated medium/large EVs. F‐ and G‐ actin fractions are positive for the EV markers Alix, annexin A2 (Ann‐A2) and negative for the Golgi and mitochondrial markers GS28 and TOM20. On the right, the histogram shows the ratio between G‐actin and F‐actin in control and CytoD‐treated large EVs (Mann Whitney test *P* = 0.05, N = 3). (c) Percentage of moving EVs from control and CytoD‐treated EVs on the neuron surface (Ctrl n = 18; CytoD n = 32; N = 10; unpaired t test, one‐tailed *P* = 0.0461). (d) Temporal analysis of calcium changes in fura‐2 loaded oligodendrocytes induced by EVs released upon ATP stimulation in the presence and in the absence of apyrase. Each trace is from a distinct oligodendroglial cell. (e) Representative calcium responses evoked by constitutively released EVs (Constitutive EVs). (f) Calcium responses evoked by the same sample of constitutive EVs kept in control condition or exposed to freeze and thaw to disrupt the EV membrane (Constitutive broken EVs) before being probed on ATP‐sensor cells. (g) Representative calcium responses evoked in oligodendrocytes by intact EVs and EVs depleted of luminal cargo. (h) Luminescence of B16‐F10 cells‐pmeLUC after 10 min exposure to vehicle (Ctrl), large EVs from 5 × 10^6^ astrocytes or 500 μM ATP. On the Right corresponding quantification of the relative pmeLUC activity expressed as photons/second (One‐way ANOVA *P* < 0.0001, Dunn's multiple comparisons test: Ctrl vs. large EVs *P* = 0.0311; Ctrl vs. ATP *P* = 0.0025; large EVs vs. ATP *P* = 0.3871; Ctrl n = 5, EVs n = 9, ATP n = 3). (i) Representative fluorescent images of quinacrine (green) positive astrocytes (top) and EVs (bottom) merged with bright field images (Scale bar = 10 μm)

Being equipped with actin filaments, as indicated by cryo‐EM (this study, Cvjetkovic et al., 2017; Zabeo et al., 2017) as well as actin binding proteins (Drago et al., 2017), a fraction of astrocyte‐derived large EVs may possess independent, active motile capability, similar to that produced by actin in cells (Cvjetkovic et al., 2017). Support to this hypothesis (Figure 6a) was provided by time lapse video recordings of single EV dynamics at the neuron surface displaying large EVs jumping from one neurite to another, as described above (Movie S4 and S5). To the best of our knowledge, there is no direct communication between the cytoplasm of adjacent neurites in differentiated neurons. Therefore, the saltatory motion could only rely on the capability of large EVs to change their shape and to interact with surface EV receptors on adjacent neurites.

Following this hypothesis, active EV movement should be sensitive to the block of actin dynamics inside EVs. Western blot analysis for F‐ and G‐ actin confirmed the presence of actin filaments in large astrocyte‐derived EVs (Figure 6b). Importantly, both F‐ and G‐actin fractions isolated from large EVs were negative for markers of intracellular organelles, thus excluding contamination with cytosolic actin. EV treatment with CytoD for 60 min caused an increase in the ratio between G‐ and F‐actin in large EVs (Figure 6b, N = 3), indicating F‐actin depolymerization, and lowered the percentage of EVs in motion at the neuron surface (Figure 6c). This suggests that actin rearrangements inside large EVs may produce motion in at least a fraction of EVs.

### 3.8 EVs contain ATP as energy source

To modify their shape and to actively move along the neuron surface, large EVs should possess an energy source that supports actin cytoskeleton rearrangements.

To assess whether large astrocyte‐derived EVs contain ATP, the main cellular energy source, we used two different bioassays previously established to measure ATP (Coco et al., 2003; Pellegatti et al., 2005). In the first assay, oligodendroglial cells, which respond to low ATP concentration (in the nM‐μM range) with rapid intracellular Ca2+ elevations (Fumagalli et al., 2011), were used as ATP sensor cells. Addition of large EVs to Fura‐2‐loaded oligodendrocytes evoked calcium transients in ∼37% of the cells (n = 84) (Figure 6d). Calcium responses were completely abrogated by treatment of large EVs with the ATP degrading enzyme apyrase (3 units/100 μl) (Figure 6d), indicating that ATP was the compound evoking Ca^2+^ transients. Constitutive large EVs, released by astrocytes in the absence of ATP stimulation, also evoked apyrase‐sensitive Ca^2+^ transients (Figure 6e, Supplementary Figure 8), excluding that externally added ATP, used to evoke EV shedding, may mediate the Ca^2+^ response in sensor cells. Given that ATP packaged inside EVs could not activate ATP receptor in sensor cells nor being degraded by apyrase, these data suggest that large EVs release or leak ATP into the extracellular fluid. To probe this hypothesis constitutive EVs were maintained in control condition or subjected to freeze and thaw, to facilitate ATP leakage from the EVs, before addition to sensor cells. Disruption of EV membrane integrity produced clear Ca^2+^ transients in sensor cells, even when responses to control EVs were barely detectable (Figure 6f). Consistently, much lower Ca^2+^ responses were evoked by large EVs, depleted of their cytosolic cargo by hypo‐osmotic shock (Gabrielli et al., 2015) (Figure 6g), revealing that ATP comes from the EV lumen. The second ATP bioassay exploited B16‐F10 cells‐pmeLUC (De Marchi et al., 2019, 2020), which stably express a chimeric firefly luciferase (pmeLUC). The chimeric protein localizes to the outside of the plasma membrane and is therefore able to sample extracellular ATP (Pellegatti et al., 2005). By this assay we could measure the ATP content of large EVs released from astrocytes stimulated with BzATP, an agonist of the P2X7 ATP receptor, which is not detected by firefly luciferase. The pmeLUC cells‐based assay confirmed that large EVs released from stimulated astrocytes contain and release ATP in the close proximity of the plasma membrane, and revealed that EVs can release ATP at concentrations in the high nM range (Figure 6h).

Finally, the presence of ATP inside astrocyte‐derived EVs was confirmed by EV labelling with 1 μM quinacrine, a fluorescence dye which stains intracellular ATP stores (Coco et al., 2003; Falchi et al., 2013) (Figure 6i). Most EVs were quinacrine positive, confirming that they store ATP (Figure 6i).

## 4 DISCUSSION

Astrocyte‐derived EVs signal to neurons via multiple mechanisms, including transfer of RNA and protein cargoes and activation of signalling events at the neuronal surface, influencing synaptic activity at both pre‐ and post‐synaptic sites (Antonucci et al., 2012; Prada et al., 2018). In addition, a role for EVs released by glial cells, especially microglia, upon ATP activation is emerging in brain diseases (Lombardi et al., 2021), such as traumatic brain injury (Liu et al., 2017) and tauopathy, where ATP‐induced EVs have been implicated in the spreading of tau protein (Ruan et al., 2020). However, how large glial EVs released upon ATP stimulation move in the extracellular space to reach target neurons and whether large EVs interact with neurons at preferential sites to influence synaptic transmission is unknown.

By using optical tweezers (OT) combined to time‐lapse imaging we hereby show that large glial EVs adhere to any neuronal compartment (cell body, dendrites, axon) and that, after adhesion, most large EVs move from the contact site scanning the neuron surface to reach preferential interaction sites in the somato‐dendritic compartment, where EVs stop moving. On the cell body, extracellular EV motion is confined to the somatic region while along dendrites and axons of developing neurons large EV motion occurs in both retrograde and anterograde direction, towards the cell soma or the periphery to reach connected neurons. EVs stop along their path especially when exploring dendritic spine protrusions on mature dendrites, resulting in lower EV speed on dendrites compared to axons in fully differentiated cultures. At preferential interaction sites EVs can elicit the formation of new filopodia. Previous studies pointed to a role for microglia in the formation of filopodia or spine head filopodia at sites of microglia–synapse contacts (Miyamoto et al., 2016; Weinhard et al., 2018). This suggests that large EVs shed from the glial cell surface retain the capacity of glial processes to induce filopodia formation at sites of persistent contact with neurons. Further work would be necessary to define whether preferential interaction points of EVs have a synaptic localization. Importantly, along developing axons, EVs move from neuron‐to‐neuron. In addition, in differentiated cultures EV speed remains elevated on axons and 50% of large EVs move in anterograde direction. This suggests that large astrocytic EVs may use axons as routes to spread their cargoes between synaptically‐coupled neurons. As the cargoes of astrocyte‐derived EVs include misfolded proteins, such as tau and amyloid‐β (Aβ) (Chiarini et al., 2017; Eitan et al., 2016), extracellular motion of large EVs along projecting axons may underlie the propagation of Aβ/tau aggregates, which occurs through an anatomically defined pattern of connections, from the entorhinal cortex up to the hippocampal region and the neocortex in the brain of Alzheimer's disease (AD) patients (Braak & Braak, 1991). Previous evidence in AD cellular models shows that small EVs storing misfolded proteins can be internalized and transferred between neurons through axonal projections (Sardar Sinha et al., 2018; Wang et al., 2017). However, intra‐ and extra‐cellular EV trafficking may be complementary, not mutually exclusive. Indeed, extracellular motion may represent a specific route to spread misfolded proteins for large EVs which exceed the axon diameter and cannot be taken up and transported intracellularly without impairing vesicle trafficking. Consistent with this hypothesis, in this study we show that the vast majority of mCLING‐labelled large EVs do not undergo internalization inside thin neurites. This may explain why large EVs move so much at the surface of thin neuronal processes. Conversely, a small fraction of large EVs contacting the cell body remained outside neurons, as proved by recapturing by optical tweezers and confocal analysis of mCLING‐labelled EVs, suggesting that transport toward the soma may be followed by endocytosis.

EVs share many features with viruses, including physical and chemical characteristics, mechanisms of biogenesis and cellular uptake (Raab‐Traub & Dittmer, 2017). Here we show that EV extracellular motion is an additional characteristic which EVs and viruses have in common. Indeed, both EVs (this study) and viruses (Burckhardt et al., 2011) are passively transported at the target cell surface by rearrangements of actin cytoskeleton. Furthermore, both virus and EV motion involves a GPI anchor protein: CAR receptor on the surface of infected cells (Burckhardt et al., 2011) and PrP on EV and, possibly, on the neuron surface (this study). In neurons, a diverse collection of receptors and ions channels are physically coupled to the actin cytoskeleton, which critically controls their localization and trafficking at the synapses (Shaw & Koleske, 2021). Like CAR receptor, PrP does not interact directly with the actin cytoskeleton. However, PrP interacts with a number of actin‐binding intracellular partners, including the synaptic vesicle protein synapsin, and the adaptor protein critical for actin‐based cell motility Growth factor receptor‐bound protein 2 (Grb2) (Nieznanski, 2010) which may couple EVs to the neuron cytoskeleton (Figure 4a). Alternatively, neuronal PrP may indirectly interact with actin via NCAM, an adhesion protein which binds to both PrP (Amin et al., 2016; Slapsak et al., 2016) (in cis) and actin filament interactors, such as Ankyrin B and ezrin (Dickson et al., 2002; Nishimura et al., 2003). Importantly NCAM, by interacting in trans with vesicular PrP (Santuccione et al., 2005), may also act as EV receptor, contributing to EV transport at the neuron surface. Further experiments are required to assess this hypothesis.

A recent work showed that PrP at the surface of brain EVs inhibits EV uptake (De Marchi et al., 2019). This is in full agreement with our study, which unveils a role for vesicular PrP in EV retention at the neuron surface and promotion of extracellular EV transport. By providing first evidence for the involvement of vesicular PrP in extracellular EV motion, our study points at PrP and its neuronal interactor(s) as potential targets to limit diffusion of EV‐associated toxic amyloid proteins in patients affected by neurodegenerative diseases.

The main accomplishment of our study is the demonstration that a fraction of EVs contains actin and has an independent capacity to move at the neuron surface, highlighting the active role of EVs in intercellular communication. EVs move on energy depleted or fixed neurons, and their motion is sensitive to block of actin dynamics by Cytochalasin D. Polymerization of G‐actin monomers at the plus (or ‘barbed’) end of an actin filament, and depolymerisation of monomers from the minus (or ‘pointed’) end can generate forces to cause movement in the EVs similar to that produced by actin in cells (Cvjetkovic et al., 2017). In support to our findings, a previous study showed that a part (albeit small) of EVs isolated from cell cultures, biological fluids or tissue displays morphological changes detectable by time‐lapse fluorescence imaging (Cvjetkovic et al., 2017). EVs could round up starting from an elongated structure, glide one along the other and stretch out flexible protrusions, morphological changes which may be induced by actin rearrangement. Active motion may be important for EVs to make their way through the limited interstitial space, which may affect extracellular EV motion in vivo. Importantly, tubular EVs which contain actin filaments strikingly resemble actin‐rich membrane protrusions such as filopodia and isolated tunnelling nanotubes (TNTs), recently characterized plasma membrane structures which assemble together to form connections between cells (Sartori‐Rupp et al., 2019). Similar to filopodia and isolated TNT, tubular EVs carry actin filaments oriented in parallel to one another, as evidenced by cryo‐EM, suggesting that EVs could derived from either budding or fission from isolated TNT/filopodia (Sartori‐Rupp et al., 2019). Future characterization of the membrane and cytoskeleton‐associated protein(s) involved in EV budding at the plasma membrane and filopodia/TNT formation may help to clarify this intriguing hypothesis.

Overall, this study provides first evidence for the existence of a regulated trafficking of EVs at the cell surface which may help EVs to reach their destination sites in vivo. Extracellular motion may be a common feature for EVs of distinct cell origin, including cancer and immune cells, and may therefore have a general role in the propagation of pathophysiological signals.

## CONFLICT OF INTEREST

The authors report no conflict of interest.

## Supporting information

Supporting Information
