# Supplementary material for: Astrocytes‐derived extracellular vesicles in motion at the neuron surface: Involvement of the prion protein
Source: J Extracell Vesicles. 2021 Jul 12;10(9):e12114. doi: 10.1002/jev2.12114 (PMC8275823; doi:10.1002/jev2.12114)
Supplement: Supplementary file 1 — Supporting Information [file JEV2-10-e12114-s001.docx]

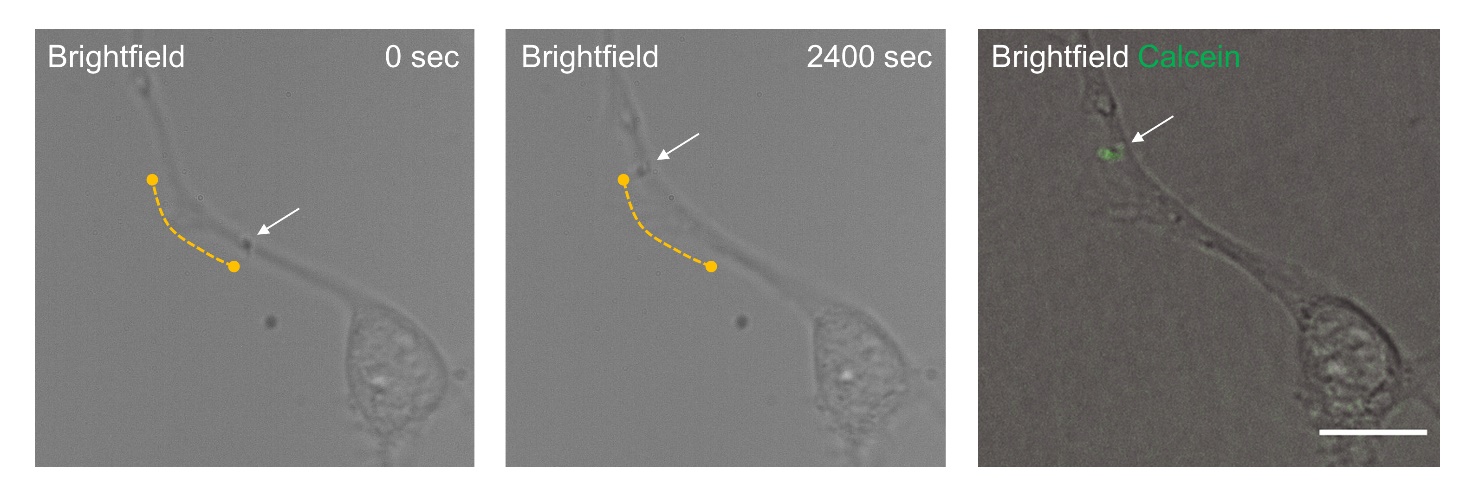


**Supplementary Figure 1a: Calcein positive EV on a neuronal process.**

Phase contrast and fluorescence images showing a green calcein positive EV (white arrow), delivered through optical tweezers, moving on a neuronal process (orange dotted line). Scale bar = 10 µm.


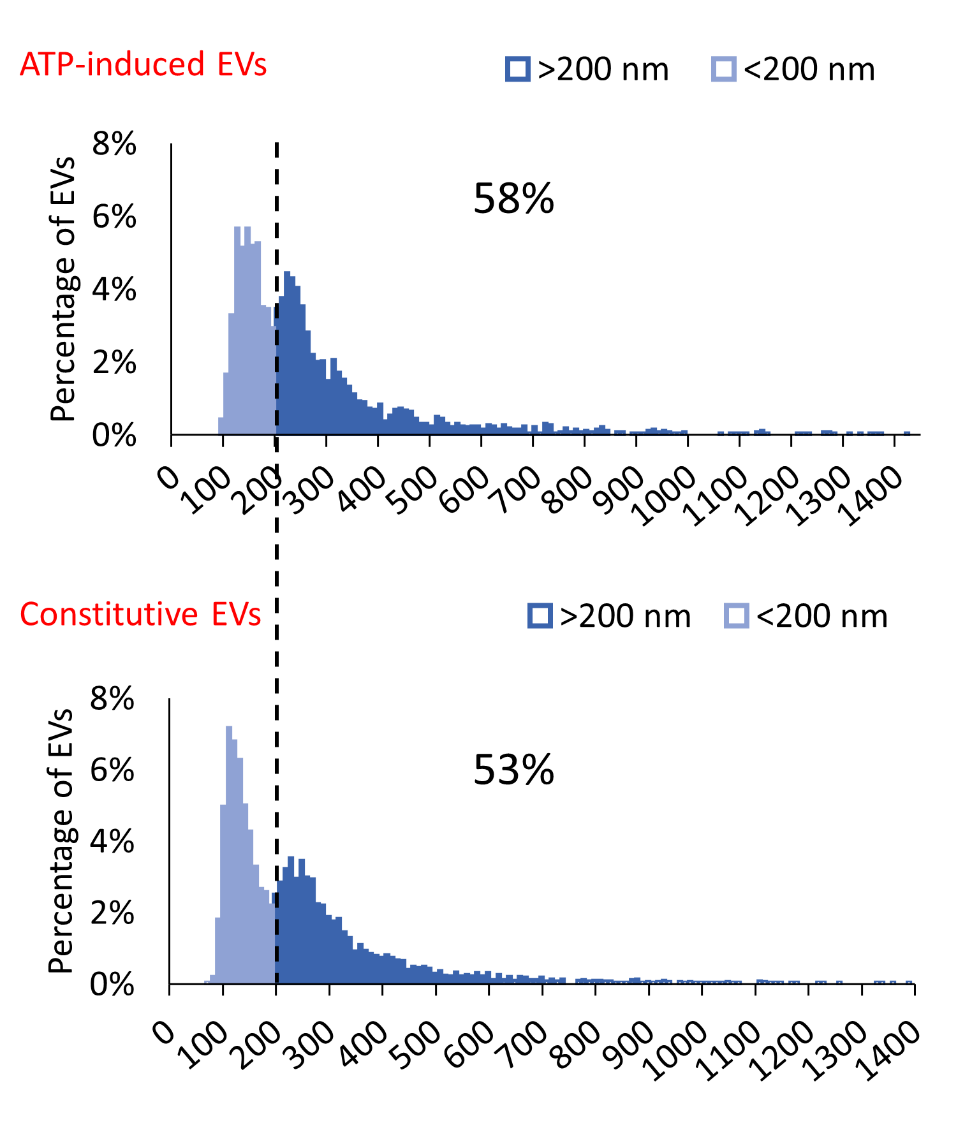


**Supplementary Figure 1b: TRPS analysis of ATP and constitutive EVs.**

Histograms show size distribution of EVs isolated from astrocytes upon ATP stimulation or constitutively released. The percentage of EVs > 200nm is similar in the two conditions: 58% for ATP-induced EVs and 53% for constitutive EVs.


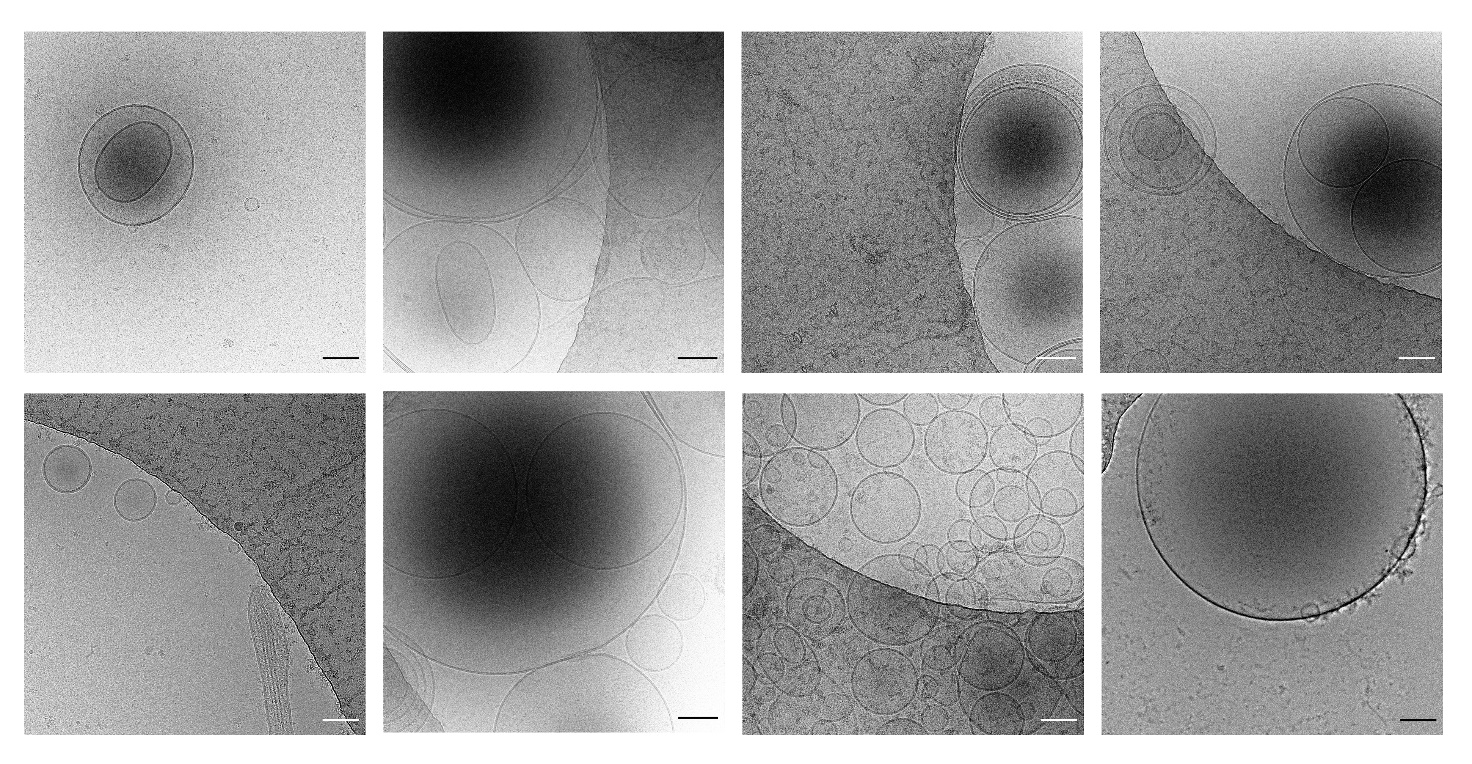


**Supplementary Figure 1c: Representative cryo-EM micrographs of EVs**

Large fields of Cryo-EM micrographs showing the medium/large EV population isolated from astrocytes at 10000x g. Scale bar = 100nm.


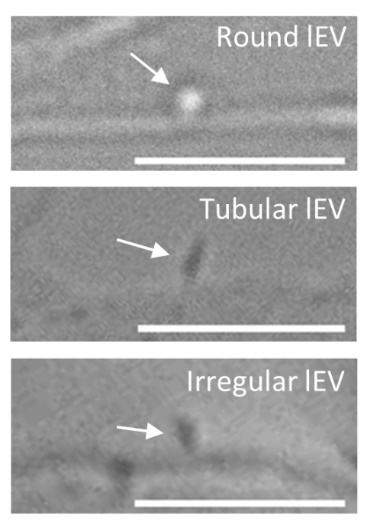


**Supplementary Figure 2: Examples of EV shapes**

Phase contrast images showing the three main EV shapes (white arrow) observed in optical manipulation experiments: Round, Tubular and Irregular EVs. Scale bar = 10 µm.


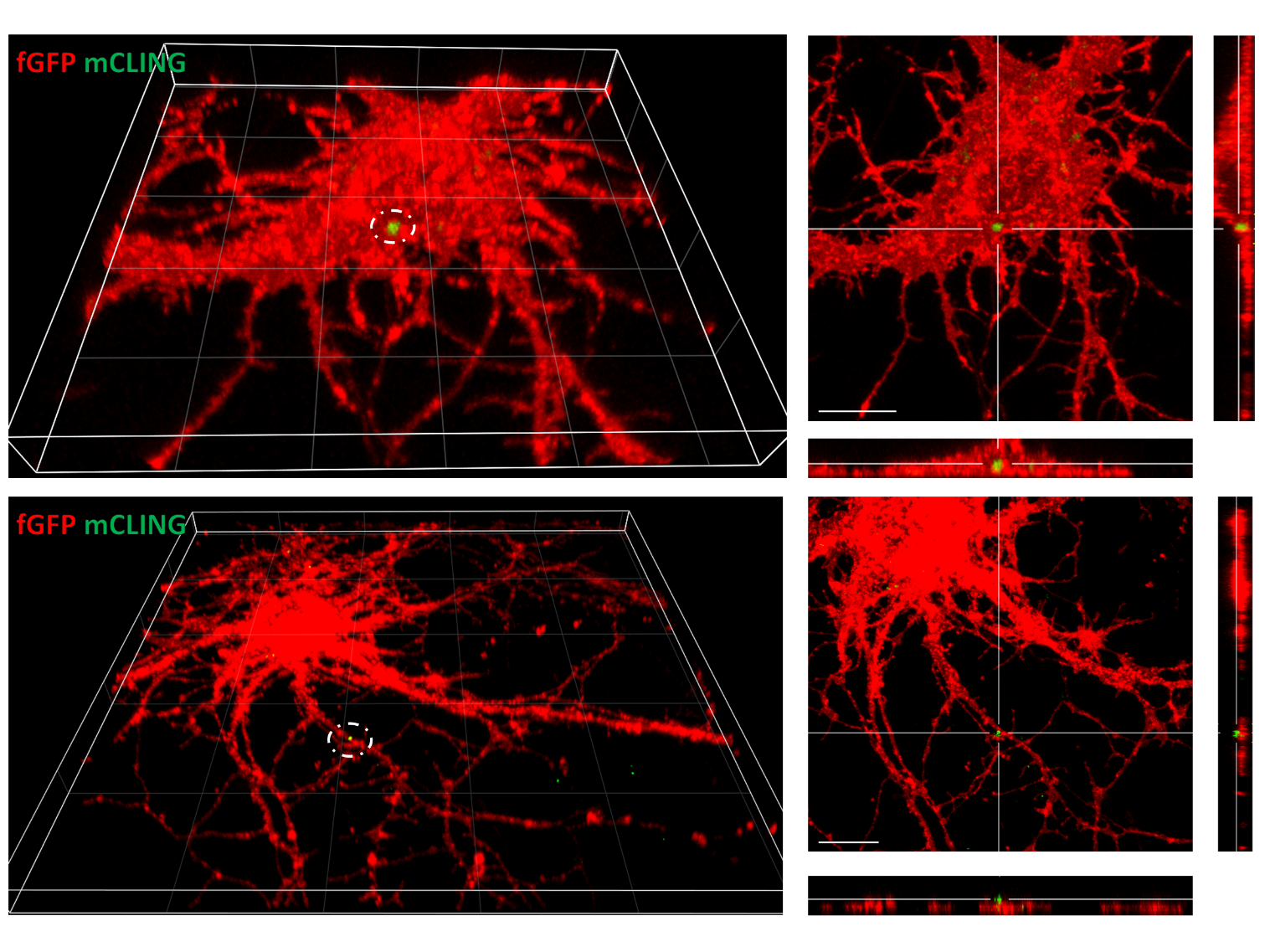


**Supplementary Figure 3: mCLING labelling of EVs.**

Confocal 3D images (left) and relative 3D projections (right) showing mCLING stained EVs (white dotted circle). The upper panel shows a mCLING EV internalized in the cell body of an fGFP transfected neuron. The lower panel shows a mCLING EV on the surface of a fGFP positive thin process.


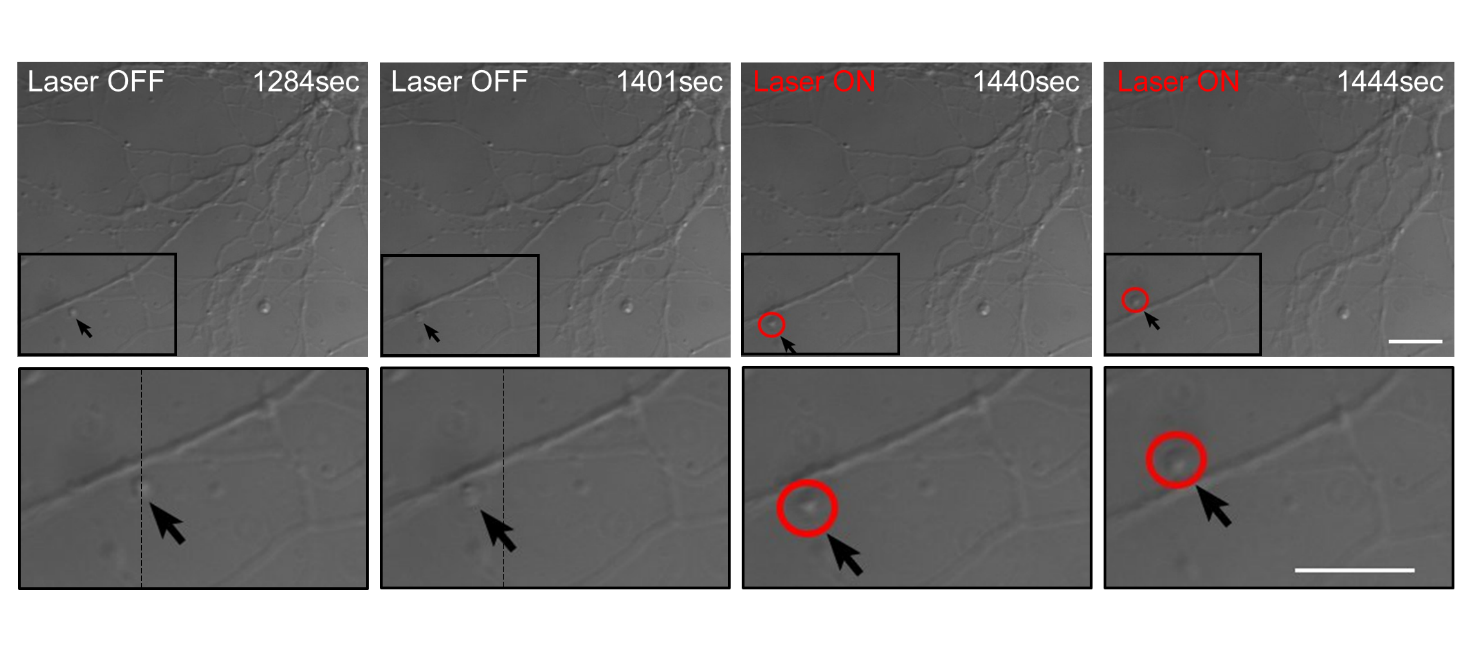


**Supplementary Figure 4: Recapturing of an EV by the laser trap**

Sequence of phase contrast images showing a moving EV (arrow) that stops upon recapturing by the laser tweezer (circle) shifting towards the trap. At higher magnification in the bottom (Scale bar = 10 µm).


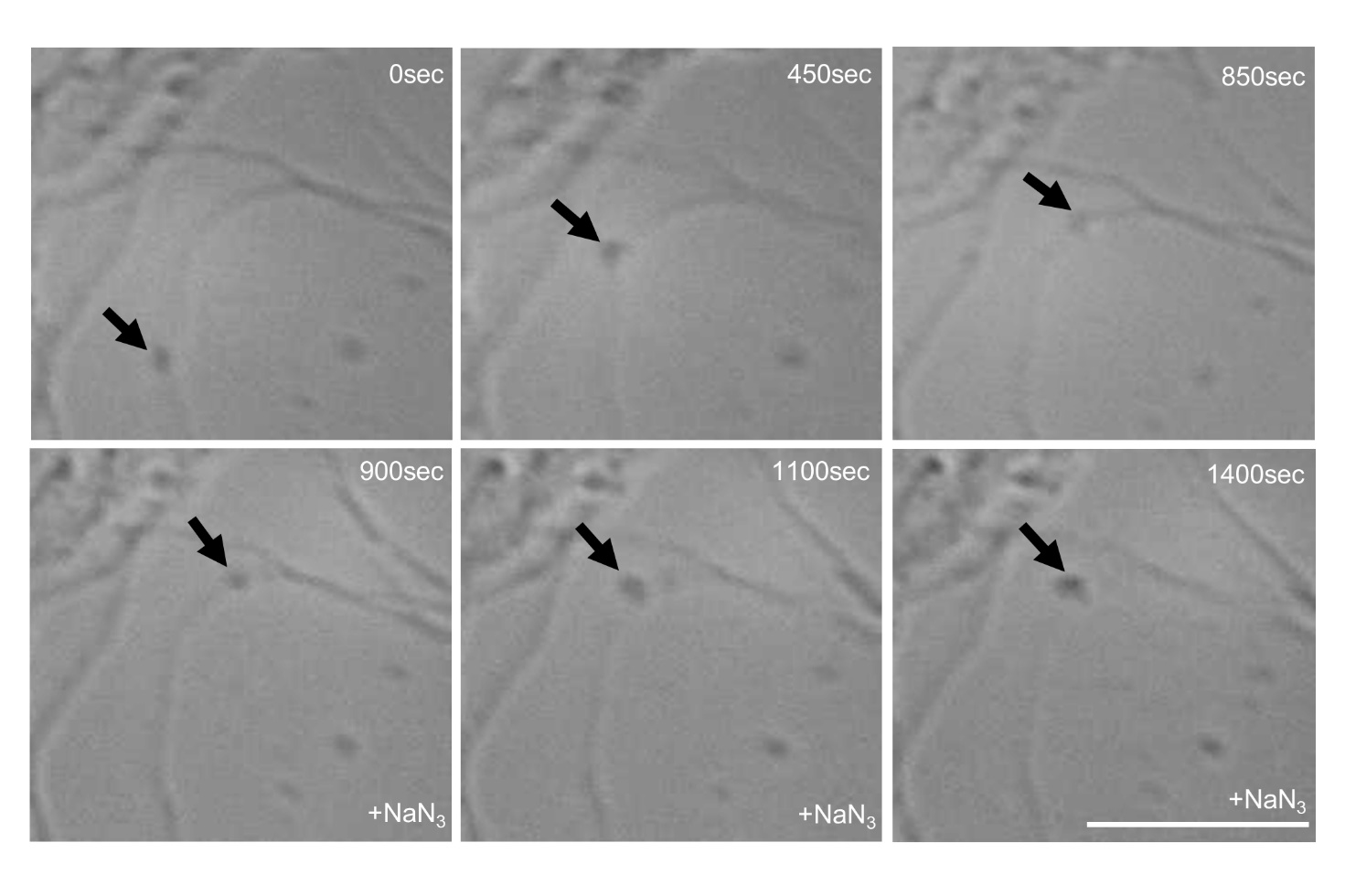


**Supplementary Figure 5: Block of EV motion mediated by Sodium Azide.**

Sequence of phase contrast images showing block of EV motion (black arrow) upon treatment with 20mM Sodium Azide (+NaN_3_) (Scale bar = 10 µm).


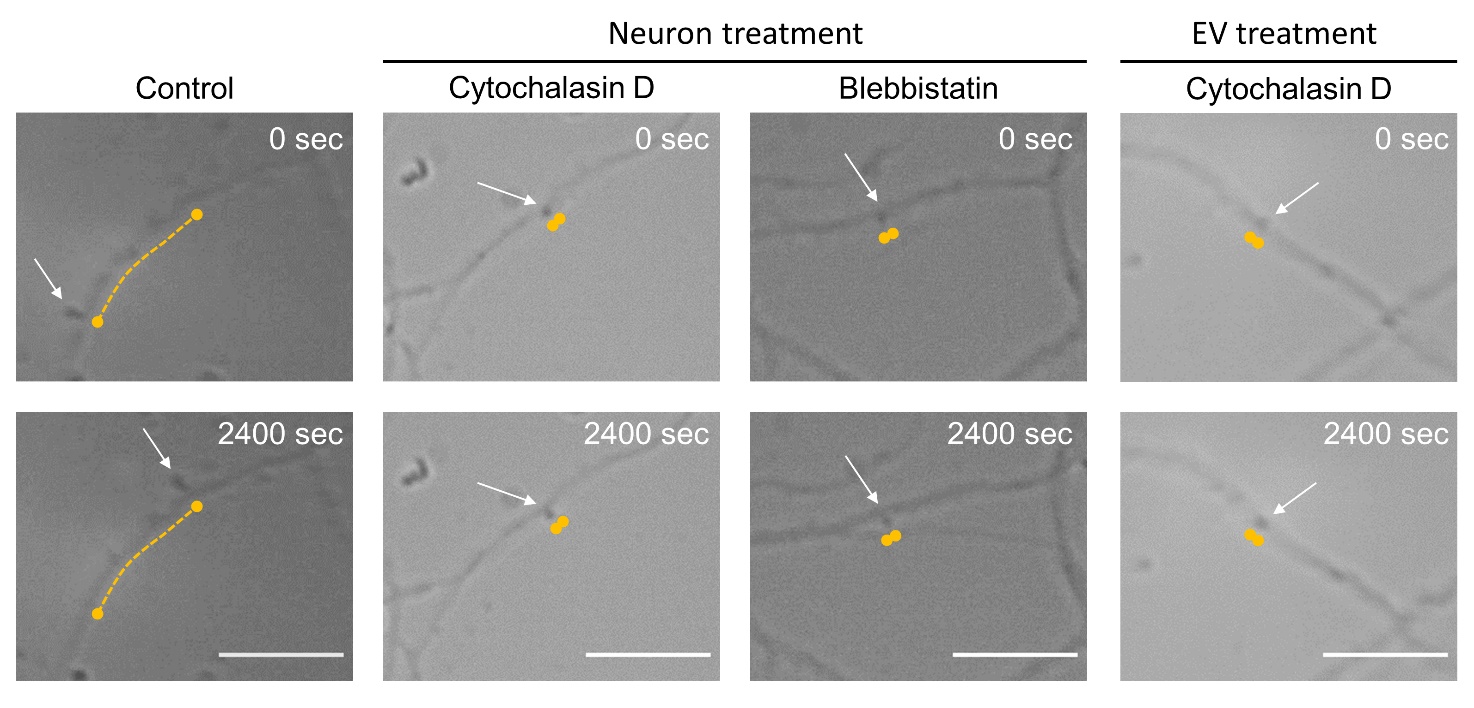


**Supplementary Figure 6: Optical manipulation of EVs after treatments with actin inhibitors.**

Phase contrast images of EVs (white arrow) delivered to neuronal processes untreated (Ctrl) or exposed to Cytochalasin D or Blebbistatin immediately after adhesion and after 20 minutes of recording. Right panels show a Cytochalasin D-treated EV positioned on a neuronal process at time 0 and after 20 minutes. Orange dotted lines highlight EV displacement. Scale bar = 10 µm.


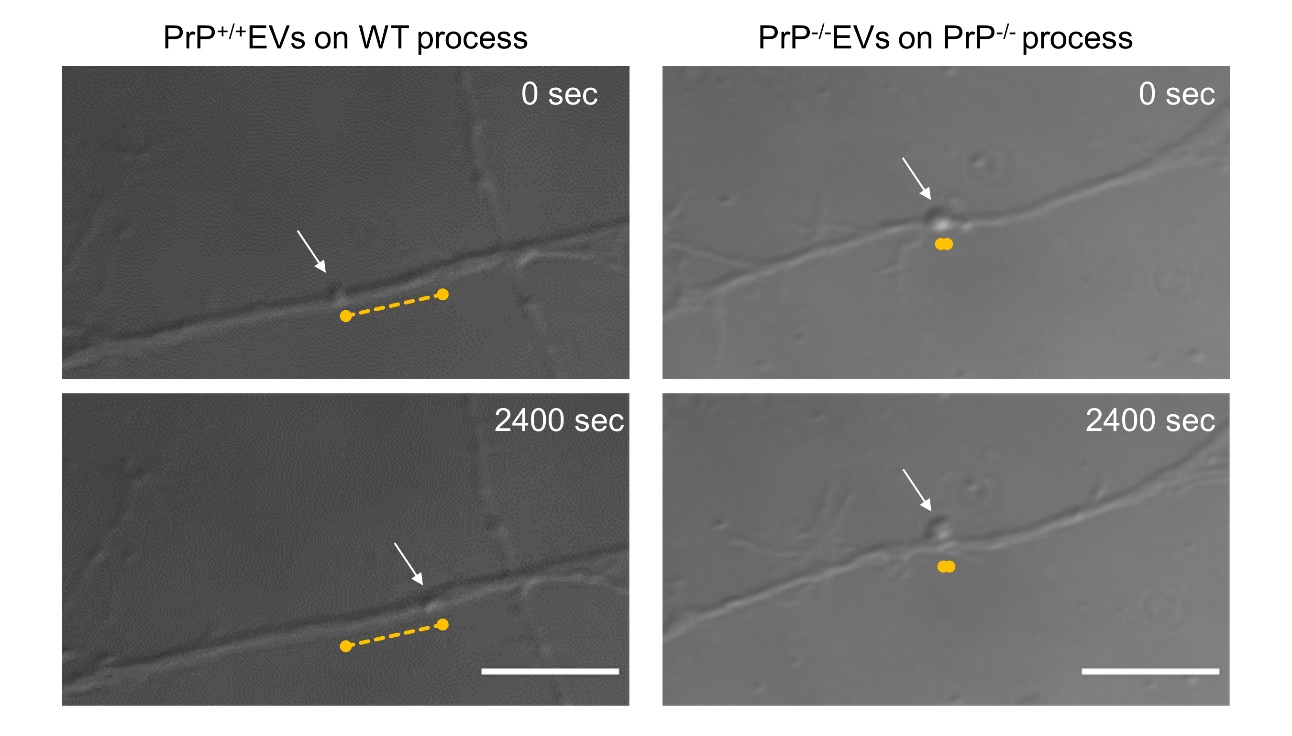


**Supplementary Figure 7: Optical manipulation of EVs delivered on WT and PrP knock out processes.**

Phase contrast images showing a moving PrP^+/+^EV (white arrow) delivered to a PrP WT process and a static PrP^-/-^EV (white arrow) placed on a PrP^-/-^ process. Orange dotted lines highlight EV displacement. Scale bar = 10 µm.


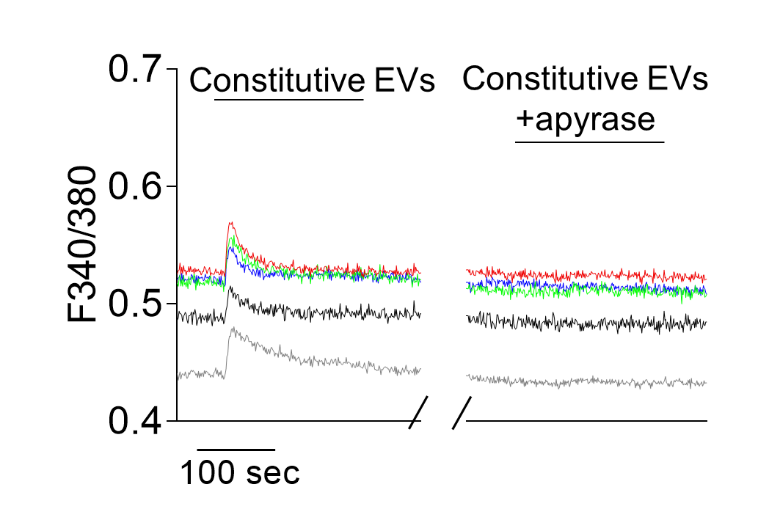


**Supplementary Figure 8:** **Calcium responses of oligodendrocytes induced by constitutive EVs.**

Temporal analysis of calcium changes in fura-2 loaded oligodendrocytes induced by constitutive EVs in the presence and in the absence of apyrase. Each trace is from a distinct oligodendroglial cell.
